# Epigenetic modifications in obesity‐associated diseases

**DOI:** 10.1002/mco2.496

**Published:** 2024-02-24

**Authors:** Yiqian Long, Chao Mao, Shuang Liu, Yongguang Tao, Desheng Xiao

**Affiliations:** ^1^ Department of Pathology, Xiangya Hospital Central South University Changsha Hunan China; ^2^ Key Laboratory of Carcinogenesis and Cancer Invasion, Ministry of Education, Department of Pathology, School of Basic Medicine Central South University Changsha Hunan China; ^3^ NHC Key Laboratory of Carcinogenesis (Central South University), Cancer Research Institute and School of Basic Medicine Central South University Changsha China; ^4^ Department of Oncology, Institute of Medical Sciences, National Clinical Research Center for Geriatric Disorders Xiangya Hospital Central South University Changsha Hunan China; ^5^ Hunan Key Laboratory of Early Diagnosis and Precision Therapy in Lung Cancer, Department of Thoracic Surgery Second Xiangya Hospital Central South University Changsha Hunan China

**Keywords:** cancer, cardiovascular diseases, epigenetics, obesity, prevention, therapy, type 2 diseases

## Abstract

The global prevalence of obesity has reached epidemic levels, significantly elevating the susceptibility to various cardiometabolic conditions and certain types of cancer. In addition to causing metabolic abnormalities such as insulin resistance (IR), elevated blood glucose and lipids, and ectopic fat deposition, obesity can also damage pancreatic islet cells, endothelial cells, and cardiomyocytes through chronic inflammation, and even promote the development of a microenvironment conducive to cancer initiation. Improper dietary habits and lack of physical exercise are important behavioral factors that increase the risk of obesity, which can affect gene expression through epigenetic modifications. Epigenetic alterations can occur in early stage of obesity, some of which are reversible, while others persist over time and lead to obesity‐related complications. Therefore, the dynamic adjustability of epigenetic modifications can be leveraged to reverse the development of obesity‐associated diseases through behavioral interventions, drugs, and bariatric surgery. This review provides a comprehensive summary of the impact of epigenetic regulation on the initiation and development of obesity‐associated cancers, type 2 diabetes, and cardiovascular diseases, establishing a theoretical basis for prevention, diagnosis, and treatment of these conditions.

## Graphical abstract

Obesity is implicated in the pathogenesis of type 2 diabetes, cardiovascular diseases, and cancers through multiple pathways, encompassing disturbances in glucose and lipid metabolism, dysregulation of sex hormones and adipokines, as well as the progression of inflammatory processes and alterations in gut microbiota. In this review, we elucidate the epigenetic modifications involved in these pathogenic mechanisms.

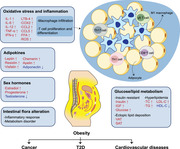

## 1 INTRODUCTION

Over the years, obesity has been regarded as an appearance problem, but evidence shows that fat accumulation and ectopic deposition are the pathogenic factors for various diseases, and there is a growing awareness that obesity is a disease in itself. The global number of obese people tripled from 1975 to 2016, and the rate continues to rise.^1^ The increase in the number of obese individuals can be attributed to a host of problems, including alterations in dietary habits, a sedentary lifestyle, and the consumption of high‐energy foods. Generally speaking, an overweight person with a body mass index (BMI) of 25 kg/m^2^ is considered overweight, and a person with a BMI of 30 kg/m^2^ is classified as obese. However, the solely use of height and weight for assessing obesity is less reliable. The inclusion of additional measures such as waist circumference, waist‐to‐hip ratio, body fat rate (BFR), visceral fat content, subcutaneous fat levels, and other indicators can enhance the precision of obesity classification.^2^, ^3^, ^4^

A large proportion of cancer cases in many regions and countries can be attributed to obesity and overweight.^5^ Compared with metabolically healthy individuals with normal weight, metabolically healthy obese individuals have an approximately 30% increased risk of obesity‐associated cancers, while the risk is even more significant for metabolically unhealthy obese individuals.^6^ In America, obesity has gradually surpassed tobacco as the leading preventable cancer cause.^7^ Prostate cancer (among males) and breast cancer (BC) (among females) have emerged as the leading types of cancer, surpassing lung cancer, as per the latest research data from the American Cancer Society. The increased incidence of BC in women is largely associated with declining fertility rates and weight gain, which also related to the rising incidence of uterine corpus cancer.^8^ In addition, obesity represents a significant risk factor for type 2 diabetes (T2D). Modest weight‐loss can mitigate T2D‐associated complications and delay the progression of the disease.^9^ Obese patients with high BFR are prone to insulin resistance (IR) and hyperinsulinemia. As the condition progresses, individuals with impaired ability to tolerate glucose can eventually develop into T2D.^10^ Cardiovascular diseases (CVDs) remain the leading contributor to the global health burden, encompassing conditions such as hypertension, atherosclerosis, coronary heart disease (CHD), heart failure, and other related ailments. Obesity and metabolic disorders not only elevate the susceptibility to CVDs but also intensify the physiological impairment caused by preexisting CVDs.^11^, ^12^

**FIGURE 1:**
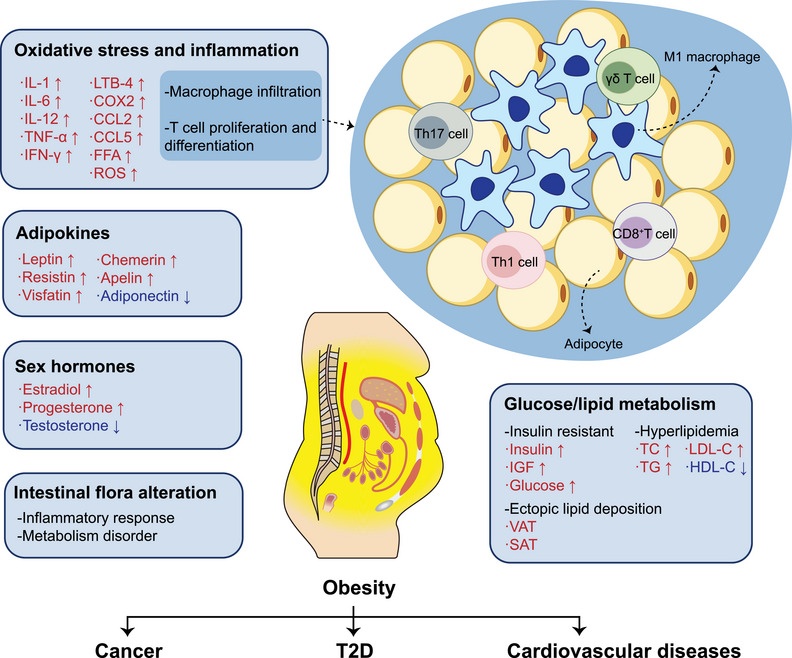
Pathogenesis of obesity‐associated diseases. Obesity is known to contribute to the development of metabolism diseases and cancers through various processes, including disorders of glucose and lipid metabolism, secretion of sex hormones, adipokines, and promotion of inflammatory responses, as well as alterations in intestinal flora. Obesity can cause oxidative stress (OS) and chronic inflammation, contributing to the release of inflammatory cytokines, chemokines, FFA, and ROS. In obesity, adipocytes organize into a crown‐like structure with surrounding infiltrated M1 macrophages, while T cells proliferate and differentiate into proinflammatory subtypes, exacerbating the progression of inflammation. The impact of AT inflammation is profound and enduring, and even lead to dysfunction in distal organs. Hyperglycemia and hyperlipidemia resulting from disrupted glucose and lipid metabolism contribute to the advancement of T2D and CVDs. Additionally, IR can hasten the growth of tumor cells. Ectopic lipid deposition can disrupt the metabolism of adjacent organs, raising the risk of developing metabolic heart disease and obesity‐associated cancers. Studies have confirmed that the increase of adipose factors, such as leptin, resistin, visfatin, chemerin, apelin, and the decline of adiponectin, are linked to progression of obesity‐associated diseases. In addition, men and postmenopausal women mainly secrete estrogen from AT, and obesity can cause an increase in estradiol and progesterone and a decrease in testosterone, which could induce reproductive system cancers. The disturbance in intestinal flora caused by obesity can also cause metabolic disorders and inflammation, which in turn can increase the risk of developing digestive cancers. Intestinal flora disorders can stimulate IR through lipopolysaccharide (LPS), and microbial metabolites can also aggravate the progression of CVDs such as hypertension and atherosclerosis. However, the above pathways interact with each other rather than work in isolation to jointly regulate the progression of obesity‐associated diseases. IL‐1, interleukin‐1; IL‐6, interleukin‐6; IL‐12, interleukin‐12; TNF‐α, tumor necrosis factor‐alpha; IFN‐γ, interferon‐gamma; LTB‐4, leukotriene B4; COX2, cyclooxygenase 2; CCL2, CC chemokines ligand 2; CCL5, CC chemokines ligand 5; FFA, free fatty acid; ROS, reactive oxygen species; IGF, insulin‐like growth factor; TC, total cholesterol; TG, triglycerides; HDL‐C, high‐density lipoprotein cholesterol; LDL‐C, low‐density lipoprotein cholesterol; VAT, visceral adipose tissue; SAT, subcutaneous adipose tissue.

Obesity can cause systemic metabolic abnormalities, and the accompanying chronic inflammatory response, endocrine changes, and the release of various cytokines are the main pathogenic factors of obesity‐associated diseases (Figure 1). In obese patients, the adipocytes hypertrophy and the abnormal lipids accumulation damage angiogenesis, causing local tissue hypoxia and necrosis, and ultimately remodeling cardiovascular system.^13^ The adipokines, inflammatory factors, and hormones released by adipose tissue (AT) can affect tumor metabolism reshaping. AT also regulate atherosclerosis and hypertension process by releasing adipokines and inflammatory factors.^14^, ^15^ Additionally, adipokines released from AT can regulate the sensitivity to insulin, while inflammatory factors and the release of free fatty acids (FFAs) can also affect glucose transport, thus promoting IR.^16^, ^17^ In addition to progressing to T2D, IR can not only increase the risk of CVDs by increasing vascular stiffness but also promote tumor cell proliferation.^17^, ^18^

The rapid increasing incidence of obesity‐associated diseases cannot be solely explained by genetics. Changes in diet and lifestyle habits can drive reversible and heritable genetic changes between cellular and individual generations through epigenetic modifications. Inconsistent with the traditional Mendelian inheritance laws, epigenetics modifies gene function without changing DNA sequences, it does fill the gap between unexplained alterations of gene expression patterns and the deletion of gene changes in pathogenesis. Various epigenetic regulatory mechanisms collaborate to uphold genetic stability, and the alterations of epigenetic regulation in individuals with obesity can potentially lead to the development of subsequent diseases.^19^, ^20^, ^21^, ^22^ Hence, conducting comprehensive research on the involvement of epigenetic modifications in the progression of obesity‐associated diseases will contribute to the prevention and therapy.

Here, we review the involvement of DNA methylation (DNAm), histone modification, noncoding RNAs (ncRNAs), and chromatin remodeling in the etiology of obesity‐associated cancers, T2D, and CVD. We also outline the pivotal role of macrophages in obesity‐triggered inflammation and the accompanying epigenetic regulation. Furthermore, dietary regulation, physical exercise, and bariatric surgery can induce epigenetic alterations to prevent and treat obesity‐associated diseases. These specific epigenetic variations could serve as biomarkers for diseases diagnosis, and the advent of epigenetic drugs also provides new therapeutic strategies for obesity‐associated diseases.

## 2 DNAm IN OBESITY‐ASSOCIATED DISEASES

**FIGURE 2:**
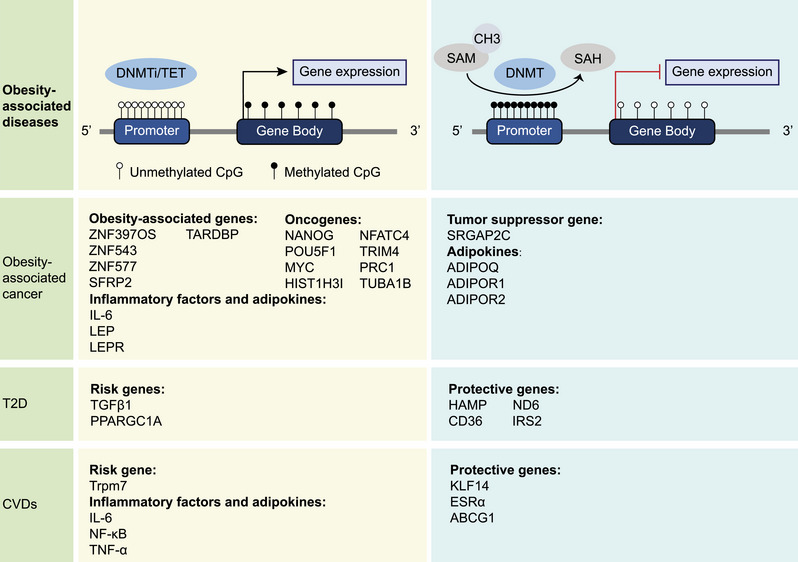
DNAm in obesity‐associated diseases. Obesity can increase the expression of obesity‐associated disease risk genes, inflammatory factors, and leptin by affecting the level of DNAm. At the same time, obesity can also reduce the expression of genes involve in self‐protective regulatory mechanism within the body. From a mechanistic perspective, DNA hypermethylation on gene promoters and enhancers can silence genes, while on the gene body can increase gene expression. In addition, the catalysis of DNA demethylases or suppression of DNMTs activity can promote DNA demethylation through active or passive pathways, respectively, and DNA demethylation on gene promoters can usually promote gene expression. SAH, S‐adenosyl homocysteine; SAM, S‐adenosyl methionine; DNMTi, DNA methyltransferase inhibitor; TET, Ten‐eleven translocation methylcytosine dioxygenase; ZNF397OS, zinc finger and SCAN domain containing 30; ZNF543, zinc finger protein 543; ZNF577, zinc finger protein 577; SFRP2, secreted frizzled‐related protein type 2; TARDBP, TAR DNA binding protein; NANOG, Nanog homeobox; POU5F1, POU class 5 homeobox 1; MYC, MYC Proto‐oncogene; HIST1H3I, histone linker 1 with Histone H3.1, NFATC4, nuclear factor of activated T‐cells cytoplasmic 4; TRIM4, tripartite motif‐containing 4; PRC1, protein regulator of cytokinesis 1; TUBA1B, tubulin alpha 1b; IL6, interleukin‐6; LEP, leptin; LEPR, leptin receptor; TGFB1, transforming growth factor beta 1; PPARGC1A, PPARG coactivator 1 alpha; Trpm7, transient receptor potential melastatin 7; NF‐κB, nuclear factor kappa B; TNF‐α, tumor necrosis factor‐alpha; SRGAP2C, Slit‐Robo Rho GTPase‐activating protein 2C; ADIPOQ, adiponectin; ADIPOR1, adiponectin receptor 1; ADIPOR2, adiponectin receptor 1; HAMP, hepcidin antimicrobial peptide; ND6, NADH‐dehydrogenase 6; CD36, CD36 molecule; IRS2, insulin receptor substrate 2; KLF14, Krüpple‐like factor 1; ESRα, estrogen receptor 1; ABCG1, ATP binding cassette subfamily G member 1.

DNAm, the common epigenetic modifications, methylates the 5th carbon atom of cytosine using the methyl provided by S‐adenosyl methionine (SAM) under the catalysis of DNA methyltransferases (DNMTs) and converts the cytosine into 5‐methylcytosine (5mC).^23^ In mammals, CpG dinucleotides are sites where DNAm usually takes place, and the areas that contain high CpG dinucleotides are called the CpG islands (CGIs).^24^ Mechanistically, obesity‐induced activities of DNMTs and demethylases can result in diverse CpG site methylation levels across regions, thereby influencing the suppression or activation of relevant gene expression^25^, ^26^, ^27^ (Figure 2).

### 2.1 Obesity‐associated DNAm and cancer development

Cancer initiation and development are mainly characterized by hypermethylation on CGIs of tumor suppressor gene promoters and global DNA hypomethylation.^28^ The expression levels of obesity‐related genes due to DNAm changes might affect cancer progression. Some overexpressed obesity‐related genes were associated with tumor‐promoting factors, while other underexpressed counterparts could inhibit cancer development.^29^ Specific CpG methylation patterns are associated with obesity.^30^ Thus, DNAm in diverse specific genes in cancers is conducive to differential diagnosis and targeted therapy. For instance, the combined methylation levels of zinc finger protein genes ZNF397OS and ZNF543 could distinguish obesity‐associated colorectal tumors from other colorectal tumors, contributing to precise medical management.^31^ Additionally, ZNF577 hypermethylation might be the epigenetic biomarker of obesity‐associated BC and is also relevant to diet patterns.^32^, ^33^ Secreted frizzled‐related protein type 2 (SFRP2) is a potential epigenetic biomarker of colorectal cancer, which could inhibit Wnt signaling. BMI could influence the DNAm level of SFRP2 in patients with colorectal cancer.^34^ Long interspersed nucleotide element 1 （LINE‐1）is often used to represent the global DNAm level, showing hypomethylation in tumors, and was considered to be statistically significant with BMI in colon cancer and BC.^35^, ^36^ Ten‐eleven translocation methylcytosine dioxygenases (TETs) are the main DNA demethylases that catalyze 5‐methylcytosine (5mC) into 5‐hydroxymethylcytosine (5hmC) and play an important role in maintaining pluripotency of stem cells.^26^ Obesity promoted the self‐renewal of triple‐negative breast cancer (TNBC) stem cells by inducing oxidative stress, which was triggered by the increased expression of TAR DNA binding protein (TARDBP) mediated by ten‐eleven translocation methylcytosine dioxygenase 1 (TET1).^37^

Obesity can increase oncogenes expression or inhibit expression of tumor suppressor genes through DNAm regulation.^38^, ^39^ For instance, obesity‐induced inflammation led to hypomethylation of the promoter regions of oncogenes such as Nanog homeobox, POU class 5 homeobox 1 (POU5F1), and MYC proto‐oncogene (MYC) in colon cells, which may eventually cause colon cancer.^40^ In tumors of obese patients with colon cancer, the promoter of oncogene histone linker 1 with histone H3.1 (HIST1H3I) is hypomethylated, while the promoter of tumor suppressor gene Slit‐Robo Rho GTPase‐activating protein 2C (SRGAP2C) is hypermethylated.^41^ Additionally, the intron of oncogene nuclear factor of activated T‐cells cytoplasmic 4 (NFATC4) demonstrates hypermethylation.^41^ The alterations in gene expression resulting from differential methylation regions will contribute to the diagnosis of obesity‐associated cancers. Nonalcoholic steatohepatitis (NASH) is highly correlated with overnutrition and IR. In some cases, patients with NASH might give rise to hepatocellular carcinoma (HCC). A genome‐wide DNAm analysis in liver tissue samples exhibited a decrease in DNAm of tumor‐related genes in NASH‐associated precancerous tissue, and this phenomenon continued and intensified in NASH‐related HCC.^42^ These overexpressed tumor‐related genes such as tripartite motif‐containing 4 (TRIM4), protein regulator of cytokinesis 1 (PRC1), tubulin alpha 1b (TUBA1B), are closely related to inflammation and malignant differentiation.

Epigenetic modifications may influence both lipid metabolism and immune response. Epigenetic research showed significantly different epigenome methylation profiles of periprostatic AT between obese/overweight and normal‐weight patients with prostate cancer. Thirty‐eight genes were found to be related to fatty acid accumulation in AT and tumor immune evasion.^43^ AT, as the primary endocrine and metabolic organ, playing a significant role in obesity‐associated cancers by secreting inflammatory factors, adipocytokines and hormones that can stimulate an adverse TME. Colorectal cancer patients with elevated DNAm levels of inflammatory factors, specifically interleukin‐6 (IL‐6), in their visceral AT (VAT) are at an increased risk of developing colorectal cancer.^44^ It is suggested that 25‐hydroxyvitamin D partly mediates this process. Various adipokines have been proven to stimulate tumor cell expansion through intracellular signal pathways.^45^ For instance, leptin exerts its effects on antiapoptotic, inflammation promotion, and angiogenesis through signaling pathways such as JAK/STAT3, MAPK, and PI3K.^46^ On the other hand, resistin promotes tumor cells proliferation, migration, and adhesion by affecting MAPK, PI3K, and NF‐κB signaling pathways.^47^, ^48^ Recent research suggested that adipokines also impact cellular communication in the cancerous process. For instance, adipose stromal cells (ASCs) could secrete adipokines to affect the gap junction loci, which affected the intercellular communication in endometrial tumors of obese patients.^49^ Mechanistically, adipokines caused promoter hypermethylation and long‐term silence of gap junction protein alpha 1 (GJA1) that encoded gap junction protein connexin‐43. In addition, organic pollutants accumulating in AT might cause an oncogenic impact through the endocrine pathway.^50^ Exposure to endocrine‐disrupting compounds might lead to intergenerational epigenetic alterations.^51^ Bisphenol A (BPA) is an endocrine disruptor with estrogenic activity and has been confirmed as a common carcinogenic chemical substance in environment that promote the progression of both hormone‐sensitive and nonhormone‐sensitive cancers.^52^, ^53^ A high‐fat diet (HFD) can increase the incidence of offspring BC induced by BPA, which is associated with DNAm‐mediated changes in gene expression.^54^ BPA can accumulate in AT, and the accumulated BPA can cause changes in lipid metabolism, further aggravating the obese state, and this vicious cycle may be one of the causes of cancer.^55^

Leptin and adiponectin secreted by adipocytes are crucial in development of cancer. In comparison with normal‐weight individuals, obese/overweight individuals get higher levels of leptin and lower levels of adiponectin in their serum.^56^ When the normal organism is in a state of hunger or reduction of body fat, the serum leptin content significantly decreases to store energy.^57^ On the contrary, the rising leptin content in response to increased body fat resists the obese state by inhibiting appetite. This negative feedback between adipocytes and the central nervous system helps maintain the lipid metabolism of the organism to keep weight. Previous research has indicated that overexpression of leptin and leptin receptors significantly affect tumor proliferation, invasion, and metastasis, accelerating the process of tumor deterioration and decrease the survival rates.^58^, ^59^, ^60^, ^61^ Intriguingly, leptin not only promotes tumor progression by regulating angiogenesis, inhibiting apoptosis, and remodeling the TME but also plays a critical role in antitumor immunotherapy through innate and adaptive immune response.^62^, ^63^ Some obese/overweight cancer patients experience better prognoses and responses to immunotherapy due to the “leptin paradox” than their normal‐weight counterparts.^62^ In untreated and newly diagnosed patients with renal cell carcinoma, promoter methylation in the leptin gene (LEP), as well as leptin receptor gene (LEPR), is remarkably higher in tumor tissues compared with normal adjacent tissues.^64^ Additionally, hypermethylation of leptin receptor gene correlated with recurrence and shorter recurrence‐free survival. In contrast, increased expression of LEP in ovarian cancer patients was related to longer lifetimes and lower recurrence rates.^65^ In addition to solid tumors, the degree of LEP methylation could also be used to predict the prognosis of acute myeloid leukemia (AML).^66^ Leptin has a dual effect on cancer progression, its antitumor immunomodulatory effects and mechanisms remain unclear. Correspondingly, due to the pleiotropic effects of leptin, its complex regulation has become an obstacle to its widespread clinical application.

Adiponectin is mainly involved in glucose and lipid metabolism, and it is known to bind to three receptors, namely adiponectin receptor 1 (ADIPOR1), adiponectin receptor 2 (ADIPOR2), and T‐cadherin. Adiponectin exerts an inhibitory effect on tumors, and promoter methylation of the adiponectin gene (ADIPOQ) is associated with cancer risk.^67^ Adiponectin can counteract the effects of leptin, exerting an antitumor effect by inhibiting angiogenesis, cell proliferation, tumor invasion, and metastasis.^68^ For patients with pancreatic cancer, treatment with the methylation‐blocking drug 5‐Azacytidine has been shown to improve the transcription level of ADIPOR1 and ADIPOR2 to enhance adiponectin signaling.^69^ This inhibitory effect on tumors by adiponectin is mainly reflected in antiproliferation. Furthermore, studies have demonstrated that chronic energy restriction can impede mammary tumor growth by altering the DNAm of LEP and ADIPOR1.^70^ Obesity was found to promote tumor progression by increasing leptin expression synergized with decreasing adiponectin expression. Meanwhile, the increased leptin levels can also directly reduce adiponectin transcription. Consequently, the alteration in leptin‐to‐adiponectin ratio plays a crucial role in linking obesity to cancer progression.^71^

### 2.2 DNAm profiles in T2D and complications

By examining the DNAm profiles of individuals with T2D, researchers have identified novel candidate genes associated with T2D and enhanced comprehension of the disease's pathogenesis.^72^ Obesity primarily leads to the development of T2D by inducing IR and disrupting lipid metabolism. Researchers have reported hypermethylation of the hepcidin antimicrobial peptide (HAMP) gene promoter in the livers of mice predisposed to T2D and obese women exhibiting IR.^73^ HAMP can serve as a candidate gene for assessing susceptibility to T2D and potentially mitigating excessive insulin secretion. CD36 plays a role in the uptake of fatty acids and the regulation of lipid metabolism. Hypermethylation and polymorphism of the CD36 promoter may be linked to the progression of obesity and T2D.^74^ In obese and T2D patients, there is an increase in CD36 promoter methylation compared with thin individuals. Additionally, reduced plasma sCD36 levels lead to an increase in low‐density lipoprotein cholesterol (LDL‐C) levels.^74^ In obese individuals, those with T2D have hypermethylation of the insulin receptor substrate 2 (IRS2) promoter CpG sites in the liver, and the reduced expression of IRS2 is closely related to IR.^75^ Among patients with T2D, the elevated expression of DNA damage‐inducible β (Gadd45β), in conjunction with TET1, stimulates the DNA demethylation of the PPARG coactivator 1 alpha (PPARGC1A) promoter, resulting in heightened gluconeogenesis and reduced glucose tolerance among patients.^76^ DNAm alterations are evident across all three stages of adipogenesis in obese individuals, and these differentially methylated genes may play a role in the development of T2D primarily by impairing the function of mature adipocytes.^77^

DNAm profiles varies among different T2D subgroups, and the initiation of diabetic complications can be predicted according to the methylation risk scores of subgroups.^78^ Advanced diabetes patients commonly experience a range of complications, increasing the complexity of treatment. Compared with normal mice, obese/diabetic mice show different expression patterns of DNMTs and TET proteins in their kidney tissues, which may promote the progression of diabetic nephropathy by regulating DNAm levels.^79^ Ten‐eleven translocation methylcytosine dioxygenase 2 (TET2) can demethylate the CGI of transforming growth factor β1 (TGFB1) to promote glomerulosclerosis and accelerating the progression of diabetic nephropathy.^80^ In addition, DNAm markers can also be used to evaluate the risk stratification of T2D nephropathy and predict the occurrence of diabetic retinopathy.^81^, ^82^ Due to technological limitations, mitochondrial epigenetic regulation has often been ignored. However, recent studies have also demonstrated the utility of mitochondrial DNA (mtDNA) methylation in the pathogenesis of IR in skeletal muscle and diabetic retinopathy.^83^ Elevated levels of FFAs in the liver can enhance the mitochondrial translocation of DNA methyltransferase 1 (DNMT1), resulting in the hypermethylation of NADH‐dehydrogenase 6 (ND6) on mtDNA.^84^ The inhibition of ND6 leads to systemic IR due to mitochondrial dysfunction.

### 2.3 DNAm and metabolic risk factors in CVDs

DNAm markers can be utilized to assess the individual's cardiac metabolic risk and offer assistance in the context of obesity‐induced CVDs.^85^, ^86^, ^87^ A meta‐analysis of genome‐wide DNAm in obese adults shows that BMI‐related CpG sites methylation with are associated with metabolic parameters of atherosclerosis and hyperlipidemia.^88^ In the whole‐genome association study, one‐third of the differentially methylated genes related to CHD are associated with obesity, among which estrogen receptor 1 ATP binding cassette subfamily G member 1 (ABCG1), and IL6 have been identified as candidate genes for CHD in multiple studies.^89^ Krüpple‐like factor 14 (KLF14) is a key metabolic‐related transcription factor, and its deficiency can accelerate the formation of atherosclerosis.^90^ The high methylation status of KLF14 is closely related to obesity indices, lipid profiles, blood pressure status, and IR status, which can provide reference for the precise treatment of cardiometabolic diseases.^91^

Abnormal secretion of adipokines is an important pathogenesis of CVDs caused by obesity. Leptin can stimulate carotid sinus nerve to increase blood pressure. The high expression of leptin and leptin receptor in carotid corpuscles is positively correlated with the promoter demethylation of transient receptor potential melastatin 7 (Trpm7).^92^ Leptin‐activated pSTAT3 can regulate the expression of Trmp7 through epigenetic modification and increase the risk of hypertension in obese patients.^92^ Inflammation is the basic feature of atherosclerosis, and the release of inflammatory factors and the recruitment of immune cells are accompanied by the whole course of the disease. The promoters of the IL‐6 and nuclear factor kappa B (NF‐κ B) genes were found to be hypomethylated in the peripheral leukocytes of obese patients.^93^, ^94^ Upregulation of IL‐6 and NF‐ κ B in leukocytes is closely associated with a decreased level of adiponectin.^93^ In obese patients, anti‐inflammatory effect of adiponectin is inhibited, leading to an acceleration of the atherosclerosis process. In addition, another study shows that the hypomethylation of IL‐6 and tumor necrosis factor‐alpha (TNF‐α) genes in obese patients can lead to endothelial dysfunction by causing inflammation and oxidative stress, thus promoting the occurrence of atherosclerosis and coronary artery disease.^94^

DNAm changes typically precede changes in gene expression, and detecting DNAm can be beneficial for early disease diagnosis.^95^ Furthermore, in obese/overweight state, DNAm regulates and reprograms gene expression patterns, which means that DNAm will become a pivotal target for treatment and drug development.

## 3 HISTONE MODIFICATION IN OBESITY‐ASSOCIATED DISEASES

Histones are involved in the formation of nucleosomes alongside DNA, and the N‐terminal end of histones can be easily modified by histone epigenetic modifying enzymes. Such modifications are associated with obesity development through the regulation of related gene expression, including methylation, acetylation, ubiquitination, phosphorylation, and others.^96^ Histone modifications occur on specific tails enriched with lysine and arginine residues, influencing gene expression through posttranslational modification. The dysregulation of these modifications can affect DNA repair, cell cycle regulation, and apoptosis, leading to the accumulation of genetic and epigenetic alterations that drive obesity‐associated complications. Here, we mainly elaborate on the role of histone methylation and acetylation in obesity‐associated diseases.

### 3.1 The impact of histone modifications on cancer‐related gene expression

Adding a different number of methyl groups to various residue sites of histone has an effect on gene expression, and different methylation degrees further complicate histone modification and regulation of gene expression.^97^ The consumption of a HFD was shown to upregulate the transcription of MYC and led to hypomethylation of histone H4K20 by Jumonji domain‐containing Histone Demethylases (JHDM) in the promoter region of the MYC regulatory genes.^98^ This leads to proliferation of prostate cancer cells and increased tumor burden. Histone methyltransferase SET domain‐containing 2 (SETD2) could catalyze H3K36me3, which is a well‐known conserved epigenetic modification.^99^ Deficiency in SETD2 was known to regulate cholesterol homeostasis and activate c‐Jun/activator protein 1 (AP‐1) via triggering lipid accumulation to promote HCC progression.^100^

Histone acetylation mainly occurs on the lysine residues in histone tails, which is a reversible dynamic equilibrium process, and the disruption of this balance has been linked to tumorigenesis. Chromatin becomes slack due to histone acetylation, thereby increasing DNA accessibility and promoting transcriptional activity. In steatotic hepatocytes, hyperacetylation of genome‐wide histones could initiate carcinogenesis by damaging DNA.^101^ Recent studies have found that the concurrent activation of acetylated and trimethylated H3K9 in mice can lead to NASH‐related HCC, and deacetylation of H4K16 promotes cancer progress by silencing genes associated with cell death.^102^, ^103^ In addition to these findings, histone modifications caused by the interactions between a HFD and intestinal microbiome are significant during gastrointestinal tumor development. Enhancer histone methylation and acetylation induced by intestinal microflora have been associated with the signal pathway of the occurrence and development of colon cancer under obesity exposure.^104^

### 3.2 Histone acetylation and methylation in initiation and progression of T2D

PPAR γ in AT can promote the growth and differentiation of adipocytes, and can reduce insulin secretion by pancreatic β cells and increase insulin sensitivity.^105^ The acetylation of PPAR γ is essential for its activation. Targeting Sirtuin 1 (SIRT1) can increase the expression levels of genes related to insulin sensitivity and ameliorate obesity‐induced T2D.^106^ Furthermore, inhibitors of histone deacetylase 3 (HDAC3) can also activate PPAR γ to reverse IR caused by HFD.^107^ Inhibition of histone deacetylases (HDACs) can also be used to prevent cardiomyopathy caused by diabetes. HDAC3 increases the H3 histone deacetylation of dual specificity phosphatase 5 (DUSP5) gene promoter in the heart of diabetic mice, which leads to cardiac dysfunction by inhibiting the expression of DUSP5. Blocking HDACs can serve as a preventive measure against diabetic cardiomyopathy. Specifically, HDAC3 stimulates H3 histone deacetylation of the DUSP5 gene promoter in the hearts of diabetic mice, resulting in cardiac dysfunction through suppressing the expression of DUSP5.^108^ HDAC3 inhibitors can also mitigate diabetic liver injury by upregulating nuclear factor erythroid 2‐related factor 2 (Nrf2), and alleviate T2D‐induced aortic fibrosis and inflammation by enhancing fibroblast growth factor 21 (FGF21) synthesis.^109^ The role of HDAC3 inhibitors in the treatment of diabetes has been widely recognized, and selective HDAC3 inhibitors can become potential antidiabetic drugs. The therapeutic potential of HDAC3 inhibitors in managing diabetes is widely acknowledged, and high selective HDAC3 inhibitors have the ability to develop into promising antidiabetic medications.^110^

In addition to histone acetylation, the modification of histones through methylation is critical in pathogenesis and progression of T2D. Premature senescence of adipocyte precursor cells and disruption in adipogenesis can lead to early IR. In adipocytes of individuals with T2D, reduced enrichment of lysine 4 tri‐methylated H3‐histone (H3K4me3) and subsequent increasing expression of mitochondrial transcription factor A (TFAM) promotes premature senescence of adipocytes, inhibits adipocyte differentiation, and accelerates the progression of T2D.^111^ Pancreatic β cells are crucial cells in the regulation of glucose homeostasis, and the defective adaptive response of β cells is one of the pathogenic mechanisms of T2D. PPAR γ agonists can upregulate the histone lysine methyltransferase SET domain containing 7 (SETD7) in β‐cells, which increases pancreatic and duodenal homeobox 1 (PDX1).^112^ PDX1 drives β‐cell adaptation to protect β cell function, thus regulating glucose sensitivity and insulin secretion.^112^

### 3.3 The dual role of histone acetylation in vascular diseases

Lysine acetylation is implicated in numerous cardiac disease processes, including cell metabolism, gene transcription, and enzyme activity, and is associated with metabolic disorders and heart failure.^113^ Histone acetylation is involved in the regulation of smooth muscle cells (SMCs) differentiation and remodels vessels, which leads to the initiation of hypertension in individuals with metabolic syndrome.^114^ Obesity‐induced hyperleptinemia may lead to histone acetylation and methylation of Trpm7, thereby promoting hypertension through upregulating Trpm7 expression.^92^ Reduced expression of the histone deacetylase Sirtuin 6 (SIRT6) in SMCs within atherosclerotic plaques may compromise telomere integrity, leading to senescence of SMCs and inflammation.^115^ Conversely, the lack of histone deacetylase 9 (HDAC9) can mitigate endothelial–mesenchymal transition (EMT) that preserve plaques stability and decelerate the progression of atherosclerosis.^116^ It is suggested that diverse HDACs may play different roles in vascular diseases.

Abnormal lipid metabolism may prompt modifying enzymes and binding proteins to recognize, produce and eliminate posttranslational modification, thereby regulating the expression of related genes and promoting the advancement of obesity‐associated diseases. With the development of mass spectrometry, additional histone acylation modifications have been discovered, which enriches the level of protein posttranslational modification regulations. Currently, various HDAC inhibitors have been approved for clinical application.

## 4 ncRNAs IN OBESITY‐ASSOCIATED DISEASES

ncRNAs refer to a class of RNAs that cannot encode proteins and are categorize into two groups: housekeeping ncRNAs and regulatory ncRNAs.^117^ Housekeeping ncRNAs are essential in fundamental cellular processes, while regulatory ncRNAs are mainly involved in modulating gene expression. Here, we will introduce epigenetic regulations of long ncRNAs (lncRNAs), micro RNAs (miRNAs), and circular RNAs (circRNAs) in obesity‐associated diseases.

### 4.1 ncRNAs in obesity‐associated diseases

LncRNAs are a type of ncRNAs lacking open reading frames and having a length greater than 200 nucleotides. They can be classified into five types: sense, antisense, bidirectional, intronic, and intergenic ncRNAs.^117^ Although lncRNAs have been regarded as “transcriptional noise” for a long time, studies have shown that lncRNAs affect cell proliferation, differentiation, apoptosis, and carcinogenic pathways.

#### 4.1.1 The impact of lncRNAs in the link between obesity and cancer

Recent research has substantiated that lncRNAs in AT act as a bridging molecule linking obesity with cancer.^118^, ^119^, ^120^ Communication between AT‐derived stem cells (ADSCs) and HeLa cells could alter the lncRNA expression profile in ADSCs, consequently enhancing the migratory potential of cervical cancer cells.^121^ In patients with obesity and diabetes, an increase in carcinogenic lncRNAs was observed in subcutaneous AT (SAT). These lncRNAs are involved in carcinogenic networks and may interact with multiple oncogenes, thereby elevating the risk of cancer.^118^ In a simulated state of excessive obesity using mature adipogenic medium, lncRNA plasmacytoma variant translocation 1 (PVT1) promoted cell epithelial‐mesenchymal transition (EMT) and enhanced cell viability and migration ability of TNBC cells by inhibiting the expression of cancer suppressor gene p21.^122^

A substantial number of lncRNAs have been demonstrated to be linked to cancer. Alterations in their expression levels and mutations have the potential to facilitate the initiation and progression of cancer. In the context of BC, lncRNA AP001429.1 can target multiple miRNAs, potentially exerting anticancer effects. Recent study has indicated that obesity can reduce the expression of lncRNA AP001429.1 in patients with BC, thus promoting cancer progression.^123^ Downregulation of lncRNA five prime to Xist (FTX) and overexpression of lncRNA small nucleolar RNA host gene 20 (SNHG20) promoted the development of nonalcoholic fatty liver disease (NAFLD) into HCC by modulating the polarization of Kupffer cells.^124^, ^125^ The leptin and estradiol levels in postmenopausal obese women are high, and the two hormones contribute to angiogenesis, survival, cell proliferation, and migration. A study of obese ovariectomized mice with a HFD suggested that the cotreatment of leptin and estradiol could increase the expression of lncRNA nuclear‐enriched abundant transcript 1 (NEAT1).^126^ This increase could induce carcinogenesis of endometrial tissue through NEAT1/mmu‐miR‐204‐5p/IGF1 axis. LncRNAs play a crucial role in predicting the prognosis of obesity‐associated esophageal squamous cell carcinoma (ESCC).^127^ Specifically, the lncRNA SLC25A21‐AS1, associated with lipid metabolism, is recognized as promoting ESCC progression via the nucleophosmin‐1 (NPM1)/c‐Myc signaling pathway.^128^ With the in‐depth investigation of genome‐wide association studies in tumor samples, the potential for lncRNAs to function as diagnostic markers and therapeutic targets for cancer has emerged.

#### 4.1.2 The role of lncRNAs in the pathogenesis of T2D

Diabetes can be categorized based on clinical characteristics, and the expression levels of lncRNAs in different clusters of diabetes vary significantly. This varied lncRNAs expression may be associated with the expression of genes involved in lipid metabolism and white blood cell fractions.^129^ The lncRNA noncoding repressor of NFAT (NRON) plays a crucial role in regulating glucose and lipid metabolism. Its deficiency has been associated with enhanced insulin sensitivity and improved serum lipid status, making it a potential target for obesity treatment.^130^ Mechanistically, deletion of NRON leads to the maintenance of lipid homeostasis through upregulating expression level of FGF21 and activating AMPK.^130^ Dysfunction of white AT (WAT) has been associated with the onset of IR, and there is an upregulation of lncRNA ADIPINT in WAT of obese individuals.^131^ ADIPINT functions by binding to pyruvate carboxylase which can modulate energy metabolism, thereby regulating lipid content and IR.^131^ lncRNA RAP2 is a white adipocyte‐selective RNA and has the ability to interact with the RNA binding protein Igf2bp2, forming a nucleoprotein complex.^132^ The LncRAP2–Igf2bp2 complex plays a crucial role in maintaining lipid metabolism homeostasis, and its decreased expression is associated with obesity‐related susceptibility to T2D.^132^ Except for the interaction with proteins, the crosstalk between lncRNA and miRNA is also one of the pathogenesis of diabetes. In the islets of obese and diabetic mice, as well as in the serum of patients with T2D, reduced expression of LncRNA Kcnq1ot1 leads to decreased levels of cyclin D1 (Ccnd1) and cyclin D2 (Ccnd2) through the targeting of miR‐15b‐5p.^133^ The absence of Kcnq1ot1 inhibits β‐cell proliferation, impairs insulin synthesis and secretion, and decreases glucose tolerance.^133^ lncRNA βFaar is markedly downregulated in the islets of obese mice, and its deletion can result in β‐cell damage and apoptosis induced by obesity.^134^ Mechanistically, βFaar upregulates islet‐specific genes by sponging miR‐138‐5p and inhibits the degradation of TRAF3 interacting protein 2 (TRAF3IP2) by binding to the SMAD specific E3 ubiquitin protein ligase 1 (SMURF1), thereby suppressing β‐cell apoptosis.^134^ In conclusion, the intricate network of lncRNAs and their interactions with various molecules and pathways have highlighted their significant role in the pathogenesis of T2D.

#### 4.1.3 Obesity‐induced lncRNA alterations on CVDs

In the obese state, lncRNAs may induce vascular endothelial dysfunction, consequently elevating the risk of CVDs. Maternal weight gain during pregnancy has been associated with reduced expression of the lncRNA KLRK1‐AS1 in offspring, leading to impaired function of endothelial colony forming cells in repairing the vascular barrier.^135^ Furthermore, exercise has been shown to downregulate the expression of lncRNA MALAT1 while increasing the levels of miR‐320a in serum of obese children/adolescents.^136^ This in turn reduces the levels of endothelial dysfunction markers such as vascular cell adhesion molecule 1 (VCAM‐1), intercellular adhesion molecule 1 (ICAM‐1), and E‐selectin.^136^ Notably, polymorphisms in the lncRNA PVT1 gene have been implicated as a potential cause of dyslipidemia.^137^ PVT1 has also been found to increase the risk of essential hypertension by modulating blood lipid levels.^137^ Moreover, a HFD has been shown to cause myocardial injury by provoking an inflammatory response and promoting cell apoptosis. Ghrelin, a polypeptide with cardioprotective properties, has been observed to target the lncRNA H19/miR‐29a/insulin‐like growth factor 1 (IGF‐1) signaling pathway and the lncRNA HOTAIR/miR‐196b/IGF‐1 signaling pathway, thereby mitigating cardiomyocyte injury associated with obesity.^138^, ^139^ These studies not only enhance our comprehension of the mechanisms by which lncRNAs regulate obesity‐associated CVDs, but also offer novel avenues and insights for future research and clinical interventions.

### 4.2 MiRNAs in obesity‐associated diseases

MiRNAs are endogenous small ncRNAs whose length is about 22 nucleotides. iRNAs repress translation through complete and incomplete base complementary pairing with mRNA, regulating the function of the target genes.

#### 4.2.1 The role of miRNAs in obesity‐associated cancers and potential therapeutic applications

The modifications of miRNAs have vital roles in diverse cancerous progress, and differential expression of miRNAs can serve as biomarkers and therapeutic targets.^140^ In HCC, miR‐148a and miR‐22‐3p could participate in tumor progression by regulating lipid metabolism.^141^, ^142^ Peroxisome proliferator‐activated receptor γ (PPARγ) is abundant in AT and known for its role in modulating energy metabolism, inflammation, adipogenesis, cell differentiation, and antitumor effect.^143^, ^144^ Obesity might decrease PPARγ by inducing overexpression of miR‐27b, miR‐130b, and miRNA‐138, which could increase the susceptibility of colorectal cancer.^145^ Obesity increases the risk of BC through various molecular mechanisms, which are partly regulated by miRNA. Upregulated miRNA‐3184‐5p and downregulated miRNA‐181c‐3p that targeted forkhead box protein P4 (FOXP4) and PPARα, respectively, could promote proliferation and invasive ability of adipocyte‐induced BC cells.^146^ Many miRNAs are now recognized as valuable diagnostic and prognostic markers for cancer, these miRNAs that play an oncogenic role are defined as oncomiRs. Chronic inflammation induced by obesity can also modulate the expression of cancer‐related miRNAs and inflammatory miRNAs (inflamma‐miRs). For instance, obese patients with BC have an increased expression of miRNA‐21 (oncomiR and inflamma‐miR) and a decreased expression of tumor suppressor miRNA‐34a compared with normal‐weight patients.^147^ In addition, miRNA‐21 and miRNA‐146a (inflamma‐miR) were significantly increased in obese patients with lymph node metastasis. MiRNA‐10b could mediate the interaction between obesity and BC in postmenopausal women by regulating cancer‐related genes.^148^ MiRNA is a promising novel noninvasive biomarker, and there are methods for detecting miRNAs in feces, blood, and urine of patients.^149^, ^150^ Endogenous mature miRNA is highly stable in serum and is not easily degraded by RNA enzymes in the blood. Research showed that miRNA‐223 in the urine of patients with endometrial cancer (EC) is evidently increasing, and there were differential expressions of miRNAs between normal‐weight and obese patients.^151^ Detecting miRNAs in body fluid will be a powerful tool for screening, diagnosis, and prognosis of cancer. However, the modification of miRNA is complex and variable. Combined detection is necessary to enhance the diagnostic value, and the differences in expression among diverse individuals may limit detection application. Thus, expanding the sample size for further study can help use miRNAs as diagnostic markers of tumors in clinical applications.

The interactions between miRNAs and adipokines play an important role in biological functions of obesity‐associated cancers. Adipokines could upregulate oncogenic miRNAs and downregulate antitumoral miRNAs, ultimately promoting tumor development.^152^ Additionally, adipokines can also affect antitumor therapy via miRNAs. Leptin could upregulate coactivator Mediator complex subunit 1 (Med1), an anchor subunit of the human mediator complex, by decreasing miRNA‐205 expression to diminish the hormonal therapy effect of tamoxifen in BC.^153^ Overexpressed miRNA‐628 could dampen the proliferation of prostate cancer cells and reduce EMT, invasive ability, and the characteristics of stem cells, while leptin can promote the development of prostate cancer by downregulating the expression of miRNA‐628.^154^ Meanwhile, miRNA‐628 could increase the sensitivity of prostate cancer cells to chemotherapeutic drugs by promoting cell apoptosis. In the case of pancreatic ductal adenocarcinoma, leptin can upregulate miRNA‐342‐3p to inhibit tumor suppressor (KLF6), which was associated with increased gemcitabine resistance.^155^ Obesity could increase leptin expression by downregulating tumor suppressor p16^INK4A^ expression, which promoted the precancerous development of BC.^156^ Interestingly, p16^INK4A^ in breast adipocytes could also mediate miRNA‐141 and miRNA‐146b‐5p to combine with leptin mRNA, negatively regulating leptin expression. Collectively, the complex interaction between miRNAs and adipokines is believed to function in the progression of obesity‐associated cancers.

**FIGURE 3:**
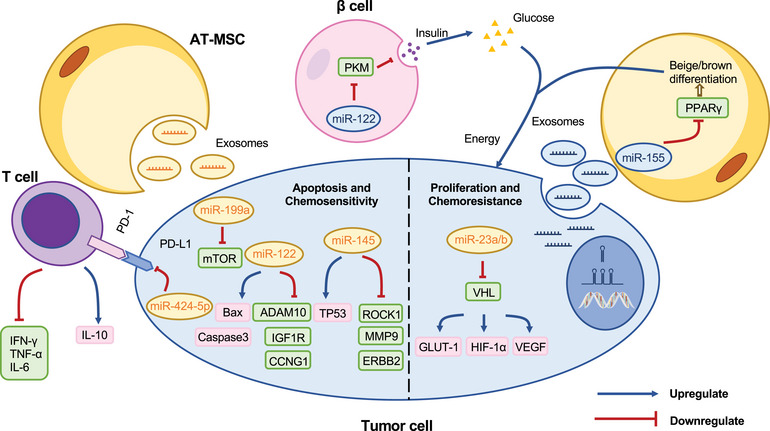
Exosomes transport miRNAs to mediate cell communication. Exosomes that are derived from AT‐MSCs have the capability to deliver miRNAs to tumor cells. These miRNAs have the ability to regulate tumor growth and sensitivity to chemotherapy. Additionally, exosomes that carry miRNAs secreted by tumor tissue have the potential to regulate insulin release from β cells and induce differentiation of adipocytes. The regulation of these physiological processes ultimately provides additional energy to tumor tissue, which further promotes the progression of cancer‐associated cachexia. AT‐MSC, AT‐derived mesenchymal stem cells; IFN‐γ, interferon‐gamma; TNF‐α, tumor necrosis factor‐alpha; IL‐6, interleukin‐6; IL‐10, interleukin‐6; PD‐1, programmed cell death protein 1; PD‐L1, programmed death‐ligand 1; TP53, tumor protein p53; PKM, pyruvate kinase M; ROCK1, Rho‐associated coiled‐coil containing protein kinase 1; MMP9, matrix metalloproteinase 9; ERBB2, erb‐b2 receptor tyrosine kinase 2; mTOR, mechanistic target of rapamycin kinase; Bax, BCL2‐associated X protein; ADAM10, a disintegrin and metalloprotease 10; CCNG1, cyclin G1; IGF1R, insulin‐like growth factor receptor 1; VHL, von Hippel‐Lindau; GLUT1, glucose transporter 1; HIF‐1α, hypoxia‐inducible factor 1 alpha; VEGF, vascular endothelial growth factor; PPARγ, peroxisome proliferator‐activated receptor gamma.

Extracellular vehicles (EVs), including but not limited to microvesicles, exosomes, apoptotic bodies, and oncosomes, are membranous vesicles that could shuttle amongst cells in the extracellular matrix, which participate in cell communication, migration, angiogenesis, and cancer cell proliferation. AT‐derived EVs involve the occurrence and metastasis of tumors and can transport miRNAs to mediate cell‐to‐cell communications^157^ (Figure 3). For example, miRNA‐23a/b was transported into HCC cells by AT‐derived exosomes and played a pivotal role in tumor proliferation, metastasis, and 5‐fluorouracil (5‐FU) resistance by targeting von Hippel‐Lindau (VHL)/hypoxia‐inducible factor 1α (HIF‐1α) axis.^158^ Instead, tumor cells could also secrete exosomes containing miRNAs that act on adipocytes. For instance, BC cells secreted exosomes containing miRNA‐155, which could induce beige/brown adipocyte differentiation and alter the surrounding adipocyte metabolism to promote cancer progression.^159^ Adipocytes differentiate into white, beige, and brown adipocytes. White adipocytes are primarily used for energy storage, while brown adipocytes can generate energy by burning fat, and beige adipocytes are an intermediate state between them. MiRNA‐155 could downregulate PPARγ expression, making adipocytes use energy more efficiently, and this high metabolic state contributed to the growth of breast tumors. In addition, tumor cell‐derived EVs could also cause abnormal glucose metabolism leading to disorders of systemic glycemic control. For instance, miRNA‐122 carried by BC cells‐derived EVs acts on pyruvate kinase M (PKM) in β‐cells, thus inhibiting insulin secretion, and contributing to the development of a hyperglycemic state that favors tumor growth.^160^ Recent studies have also suggested that the specific miRNome profile of platelets in patients with visceral obesity was involved in the initiation of colon cancer, and the regulation of platelet activation may become another breakthrough in the therapy of obesity‐associated cancers.^161^

MiRNAs are direct or indirect factors in cellular communication between adipocytes and tumor cells^162^, ^163^; thus, utilizing the transport function of exosomes may provide a new direction for cancer treatment. Scientists have leveraged AT‐derived exosomes to transport miRNAs to tumors which triggers apoptosis of cancer cells and enhances the sensitivity to chemotherapy (Figure 3). Here, miRNAs downregulate the expression of target genes by binding to the mRNA of carcinogenic factors. For example, AT‐derived mesenchymal stem cells (AMSCs) secrete exosomes with miR‐122 to suppress the expression of a disintegrin and metalloprotease 10 (ADAM10), insulin‐like growth factor receptor 1(IGF1R), and cyclin G1 (CCNG1) in HCC cells, resulting in arrest of cell cycle and apoptosis, and improve chemotherapy efficacy.^164^ AMSC‐derived exosomes can delivery miR‐145 to BC cells to prevent cancer progression by suppressing the expression of Rho‐associated coiled‐coil containing protein kinase 1 (ROCK1), matrix metalloproteinase 9 (MMP9), erb‐b2 receptor tyrosine kinase 2 (ERBB2) and upregulation of tumor protein p53 (TP53).^165^ Similarly, miR‐199a enhances liver cancer sensitivity to doxorubicin by inhibiting the mTOR pathway.^166^ MiR‐424‐5p restrained the expression of programmed death‐ligand 1 (PD‐L1) on TNBC cells, which create an inflammatory milieu.^167^ AMSC‐derived exosomes, serving as endogenous carriers, exhibit stability and low immunogenicity, rendering them an innate vehicle for miRNAs transportation. They can effectively counteract the drawbacks of instability and susceptibility to degradation of exogenous miRNAs. Despite the promising prospects, exosomes still face technical impediments in terms of separation, identification, and commercialization, and their clinical applications remain in the nascent stage.

#### 4.2.2 MiRNAs as potential biomarkers and therapeutic targets for T2D

The fact that miRNAs can be used as markers to predict the risk of T2D is significant in early disease detection and intervention. The study of plasma miRNA in preadolescent children provides valuable insights into the potential biomarkers associated with obesity and IR.^168^ In this research, miR‐18a‐5p, miR‐146a‐5p, miR‐152‐3p, and miR‐423‐3p could potentially serve as important indicators for identifying individuals at risk for developing T2D.^168^ Upregulation of miR‐155, miR‐29, and miR‐548ag is observed in T2D patients, and targeting these miRNAs may decrease fasting plasma glucose levels and improve glucose tolerance.^169^, ^170^, ^171^ AT serves as the primary origin of serum exosomes that can serve as indicators of AT function and potential prognostic markers for T2D. In obese patients with T2D, there is an elevation in hsa‐miR‐551b‐3p levels in both VAT and serum exosomes compared with obese patients without T2D, suggesting a potential association between miRNAs secretion and obesity‐induced lipid remodeling.^172^ Furthermore, miR‐27‐3p overexpression in exosomes originating from M1 macrophages results in impaired mitochondrial autophagy through the suppression of ras homolog family member T1 (Rhot1) gene, which encodes mitochondrial rho GTPase 1.^173^ Mitochondria dynamics are believed to contribute to the advancement of diabetes, as autophagic impairments within the mitochondria can exacerbate oxidative stress in β cells and foster IR.^174^ Consequently, the induction of mitochondrial autophagy holds promise for potential therapeutic benefits in the treatment of diabetes.

MiRNA plays a key role in diabetic nephropathy, diabetic retinopathy, and diabetic heart disease, and can be used as a new marker and therapeutic target for the prevention and treatment of diabetic complications in the future. Rab3A/27A system is an important protein family involved in extracellular vesicles secretion, which affects intercellular communication by regulating the transport of miRNAs in vesicles and participates in podocyte injury in diabetic nephropathy. Under obese status, miR‐223‐3p leads to high blood glucose, abnormal insulin release, abnormal development of the ocular vascular system, and diabetes with retinopathy. The expression of miR‐340‐5p is significantly increased in heart tissue of diabetic patients, targeting myeloid cell leukemia 1 to induce mitochondrial dysfunction, worsen oxidative stress, and play a crucial role in the development of diabetic cardiomyopathy.

#### 4.2.3 MiRNAs as predictive biomarkers for obese patients with CVDs

MiRNAs can be utilized to predict the early onset of CVDs in obese patients. In the baboon model of early atherosclerosis, changes in the miRNA expression profile were observed, and a significant correlation was found between miR‐5001 and miR‐7975 and lesion load.^175^ However, further exploration is required to elucidate the specific mechanism. Furthermore, in comparison with healthy individuals, patients at high risk of CVDs exhibited varying degrees of increased plasma miR‐182‐5p, miR‐199a‐5p, miR‐193a‐5p, and miR‐155‐5p.^176^ Due to chronic inflammation resulting in myocardial damage and reduced cardiac contractility, obese patients are susceptible to left ventricular dysfunction. Consequently, the expression levels of miR‐101‐3p, miR‐140‐3p, and miR‐99a‐5p in the plasma of obese patients can serve as predictive markers for early myocardial injury.^177^ Patients with coronary artery disease exhibit distinct miRNA expression profiles in both epicardial and VATs compared with healthy individuals.^178^ The differential expression of certain miRNAs in these ATs is associated with varying clinical outcomes. Exosomes derived from visceral fat carry miR‐27b‐3p into endothelial cells, thereby targeting the PPAR α/NF‐ κ B pathway.^179^ This mechanism can exacerbate atherosclerosis by promoting inflammation.

### 4.3 CircRNAs in obesity‐associated diseases

CircRNAs are characterized by their closed loop shape that lacks 3′ polyadenylation (poly(A)) tails and 5′ cap, making them stable and resistant to degradation. circRNA, as a competing endogenous RNA (ceRNA), could competitively combinate with miRNA to attenuate negative regulation of downstream target genes and increase target gene expression.^180^ Although the primary function of circRNAs is to act as miRNA “sponges” in regulating target gene expression, some circRNAs could also exert their functions by interacting with proteins.^181^ CircRNAs have been confirmed to be involved in lipid synthesis and decomposition, and its imbalance can lead to a series of metabolic diseases.^182^, ^183^

CircRNAs play a dual role in regulating inflammation in AT. For instance, circRNAs participated in regulating inflammation response through an obesity‐associated ceRNA network, which was related to lipid metabolic disorders.^184^ On the contrary, circARF3 (ADP‐ribosylation factor 3) could also strengthen mitophagy to subdue adipose inflammation by combining miRNA‐103.,^185^ but whether changes in this inflammatory state affect tumor development have not been confirmed. A recent study identified circRNAs profiles in white and brown AT, which provided circRNA candidates for regulating adipose formation.^186^, ^187^ Studies suggested that circSAMD4A increased preadipocyte differentiation,^188^ and circArhgap5‐2 played an essential role in adipogenesis and lipid metabolism.^189^

Evidence suggests that downregulated hsa_circ_000839 in patients with BC exhibits a negative correlation with BMI, but the underlying mechanism is still unclear.^190^ A transcriptome study of circRNAs was conducted, focusing on obese and postmenopausal patients with EC and revealed the presence of 174 differentially expressed circRNA in EC tissues compared with adjacent normal tissues.^191^ Nonetheless, additional functional research is required to determine the circRNAs that can offer diagnostic and prognostic assistance in relation to obesity‐related EC. The plasma of TNBC patients with high BFR showed increased expression of exo‐cirCRIM1 derived from adipocytes.^192^ Exo‐cirCRIM1 can increase protein glycosylation of fructose‐bisphosphatase 1 (FBP1) by suppressing miR‐503‐5p and thus weakening its glycolytic inhibition, which promotes TNBC progression. While circRNAs were confirmed to contribute to obesity and cancer development, whether circRNAs play a part in the interaction between obesity and cancer has been rarely explored.

SIRT1 is involved in regulating insulin sensitivity and oxidative stress, and it plays a protective role in T2D.^193^ In T2D patients, the expression of hsa_circ_0115355 was abnormal, and the reduced hsa_circ_0115355 hindered its competitive binding with miR‐145, resulting in decreased expression of SIRT1.^193^ The upregulation of circ LRP6 in damaged β cells leads to increased apoptosis, decreased insulin release, and induction of oxidative stress, exacerbating T2D by targeting the miR‐9‐5p/protein N‐arginine methyltransferase‐1 (PRMT1) signaling pathway.^194^ Forkhead box O1 (FOXO1) is a negative regulator of insulin signal and plays an important role in the regulation of blood glucose.^195^ Oleic acid can promote the activation of circ HIPK3, leading to increased fat deposition and IR by sponging miR‐192‐5p and upregulating FOXO1.^196^

Due to their tissue specificity and conservation, circRNAs can serve as potential biomarkers for diagnosing coronary artery disease.^197^ For instance, in comparison with healthy individuals, the expression of hsa_circ_0124644 was significantly upregulated in the peripheral blood of patients with coronary artery disease and validated in larger sample sizes.^198^ Mechanistically, circRNAs can competitively bind to miRNAs to regulate the expression of genes associated with coronary artery disease.^87^ CircRNAs derived from extracellular vesicles can also elevate the risk of hypertension and atherosclerosis by modulating blood lipids, lipid deposition, and endothelial cell function.^199^

Evidence shows that ncRNAs have significant biological functions and participate in forming complex gene regulatory networks. Exosomes assist in transporting ncRNAs to distant organs, amplifying the negative effects of AT. These molecules interact with each other, facilitate intercellular communication and collectively contributing to the progression of obesity‐associated diseases.

## 5 CHROMATIN REMODELING IN OBESITY‐ASSOCIATED DISEASES

Changes in the position and structure of nucleosomes give rise to alterations in gene transcriptional activity controlling gene expression, which is called chromatin remodeling. Histone covalent modification and ATP‐dependent chromatin remodeling are two types of chromatin remodeling. According to the unique domains found in catalytic ATPases and associated subunits, chromatin remodeling complexes are categorized into four subfamilies, namely: switching defective/sucrose nonfermenting (SWI/SNF), imitation switch (ISWI), chromodomain helicase DNA‐binding, and inositol requiring 80 (INO80) family remodelers.^200^ These ATP‐dependent remodelers exert a DNA translocation mechanism to achieve nucleosome assembly, chromatin access, and editing of nucleosome.^201^ The energy produced by ATP hydrolysis powers the “sliding” of nucleosomes on DNA. Remodelers could expose or cover binding sites at promoters and enhancers of the gene via a loose and tight nucleosome arrangement, which led to gene silencing or expression.^201^ Adipocytes can provide energy to ATP‐dependent chromatin remodeling complexes, the accessibility of chromatin with transcription factors increases, and gene transcription starts.

Compared with healthy‐weight patients, there is an enrichment of chromatin modification and remodeling genes in tumor tissues of obese patients with prostate cancer, which provides a potential connection between obesity and cancer progression.^202^ Chromatin remodeling protein scaffold/matrix attachment region‐binding protein 1 (SMAR1) was known for its role in recruiting HDAC1/mSin3a complex to PPARγ promoter to attenuate PPARγ expression level, which had a negative effect on adipocytes differentiation and adipogenesis.^203^ Apart from this, SMAR1 was also proved to be a suppressor in tumor progression.^204^ The inhibitory effect of SMAR1 on adipogenesis and tumorigenesis might provide a novel thread to obesity and obesity‐associated cancer. AT‐rich interactive domain‐containing protein 1A (ARID1A), a DNA‐binding component of the SWI/SNF chromatin‐remodeling complex, was corroborated to play a crucial role in the pathogenesis of NASH.^205^ In addition to contributing to steatohepatitis, loss of ARID1A has epigenetic effects on development of established HCC. While overexpression of ARID1A promoted tumor initiation, loss of ARID1A could be discovered in established tumor that degraded chromatin accessibility and downregulated expression of genes relative to tumor progression.^206^ Transcriptional corepressors SWI/SNF complex could be recruited by nuclear receptor Nur77 dampening expression of CD36 and fatty acid‐binding protein 4 (FABP4) and subsequently inhibited uptake and transport of fatty acid, reducing the energy source necessary for BC development.^207^ The chronic stimulation of obesity can result in cellular adaptation, leading to the differentiation of cancer cells into stem cell‐like characteristics. Mechanistically, prolonged exposure to obesity enhances the chromatin accessibility of the transcription factor CCAAT/enhancer‐binding protein beta (C/EBPB), thereby promoting the increased expression of downstream genes associated with cancer dedifferentiation.^208^

Inflammatory stress can lead to β cells damage, which constitutes one of the pathogenic processes in diabetes. The Vitamin D receptor shuttles between chromatin remodeling complexes BRG1‐associated factors (BAF) and polybromo‐associated BRG1‐associated factors (PBAF), regulating nuclear chromatin accessibility to protect β cells through the modulation of anti‐inflammatory protein expression.^209^ In obese/diabetic mice and diabetic patients, the downregulated expression of DNA binding protein CCCTC‐binding factor (CTCF) in β cells leads to dysregulation of chromatin accessibility.^210^ Dietary intervention can restore CTCF expression and protect β cell function and maintain glucose homeostasis through chromatin remodeling of genes related to glucose metabolism and stress response.^210^ Poly (ADP‐ribose) polymerase 1 (PARP‐1), a DNA binding protein, plays a role in DNA repair and chromatin remodeling, and is activated in diabetic cardiomyopathy. Mechanistically, PARP‐1 targets the SIRT1/peroxisome proliferator‐activated receptor gamma coactivator 1‐α (PGC‐1α) axis, leading to increased oxidative stress, inflammation, and fibrosis in myocardium. BAF60a is a subunit of SWI/SNF chromatin remodeling complex which is an important regulator of adipose inflammation induced by obesity.^211^ In obese and T2D mice, macrophage infiltration in AT was associated with decreased expression of BAF60a in stromal vascular fractions.^211^ The abnormal expression of BAF60a and its downstream effector activating transcription factor 3 (ATF3) promotes the release of inflammatory cytokines and aggravates the T2D process.^211^

Although chromatin remodeling in obesity‐associated diseases has recently attracted research interest, this field is relatively young.

## 6 EPIGENETIC MODIFICATIONS OF MACROPHAGES IN OBESITY‐INDUCED INFLAMMATION

It is widely recognized that immune regulation plays a critical role in obesity‐induced inflammation in AT. Hyperplastic AT, often accompanied by abnormal immune cell infiltration and continuous chronic low‐level inflammation, can not only cause obesity‐related metabolic complications such as IR and CVDs but also promote the development of cancers.^212^, ^213^ Infiltration of macrophages in AT of obese patients increased from under 10% in the normal state to nearly 40%, and the phenomena of macrophage infiltration were more prominent in VAT.^214^, ^215^ With an increase in lipid accumulation, hypertrophic adipocytes are encircled by macrophages, resulting in the formation of crown‐like structures (CLS), which are associated with a negative impact on cancer prognosis.^216^, ^217^ Macrophages can be classified into two phenotypes: M1 (proinflammatory state) and M2 (anti‐inflammatory state).^218^ Diet‐induced obesity was confirmed to convert macrophages to M1 polarized chronic inflammatory condition in AT.^219^ Here, M1 macrophages can produce cytokines such as TNF‐α and IL‐6 to promote progression of obesity‐associated diseases.^220^ Considering that macrophage polarization is largely influenced by environment, the role of epigenetic modifications in this area has attracted scholars’ attention.

Obesity mainly causes an increase in the infiltration of M1 macrophages. The cytokines secreted by M1 macrophages can accelerate lipid metabolism disorder, chronic inflammation, cell apoptosis, and other biological processes in AT, which is particularly common in metabolic diseases. For instance, in animal models with a HFD, lipotoxic hepatocytes‐derived extracellular vesicles released miR‐192‐5p and miR‐9‐5p to facilitate M1 macrophage activation, promoting the development of NAFLD.^221^, ^222^ Conversely, M2 macrophages can suppress obesity‐induced inflammation and hinder the advancement of cardiovascular metabolic disorders. Research has demonstrated that exosomes containing IL‐4 can stimulate macrophages to differentiate into anti‐inflammatory phenotypes and enhance glucose uptake by upregulating PPAR γ.^223^ Administering exosomes with IL‐4 via intraperitoneal injection to obese mice can reprogram circulating inflammatory signals and mitigate the inflammatory response of cardiovascular system.^223^

Central obesity is a leading risk factor for IR, and IR is also the most crucial link in the carcinogenesis mechanism that is associated with the decline of glucose uptake and utilization rates. HFD could lead to M1 polarization of bone marrow macrophages (BMMs), and the elevated miR‐143‐5P derived from BMMs could induce IR in hepatocytes.^224^ In addition, high expression of miR‐27‐3p in M1 macrophages induced by a HFD leads to the disruption of mitochondrial autophagy, which exacerbates IR.^173^ Berberine is known to alleviate obesity, IR and liver injury by inhibiting macrophages from releasing miR‐155‐5p, which protects the liver from lipotoxicity.^225^ Another study suggested that berberine could also partly inhibit lncRNA Gomafu expression from suppressing obesity‐induced inflammation through M2 polarization of macrophages.^226^ Downregulation of lysine (K)‐specific demethylase 2A (Kdm2a) increased the H3K36me2 level and enhanced chromatin accessibility, which promotes the conversion of macrophages to an anti‐inflammatory state.^227^ Silencing of Kdm2a effectively mitigated the progression of both IR and obesity. Additionally, employing miRNA to regulate the differentiation and proliferation of protective subsets of lipid‐associated macrophages has the potential to prevent obesity‐induced metabolic disorders.^228^ M2‐like macrophages are capable of maintaining metabolic homeostasis, and overexpressed miR‐690 in M2 bone marrow‐derived macrophages enhanced insulin sensitivity both in vivo and in vitro, which might also be available for metabolic therapy of metabolic diseases.^229^

The relationship between chronic inflammation and cancer has been confirmed. Inflammation in the AT microenvironment can lead to changes in metabolic syndrome, which is believed to be associated with cancer risk and progression.^230^ Macrophages comprise the primary constituent of abnormally infiltrated immune cells in the vicinity of AT and are major participants to the chronic inflammation induced by obesity. In addition to macrophages, obesity has an impact on numerous other immune cells. As an example, prolonged chronic inflammation overstimulates T cells, leading to T cell exhaustion and increased PD‐1 expression, which ultimately promoted tumor growth.^231^ This suggests that obese patients may be more responsive to immune therapy. In general, the changes in immune cells and inflammation caused by obesity play an important role in the process of tumor development. Intervention of AT inflammation may become a new strategy for the treatment of obesity‐associated diseases and epigenetic modifications will contribute to this strategy.

## 7 PREVENTION AND TREATMENT OF OBESITY‐ASSOCIATED DISEASES

**FIGURE 4:**
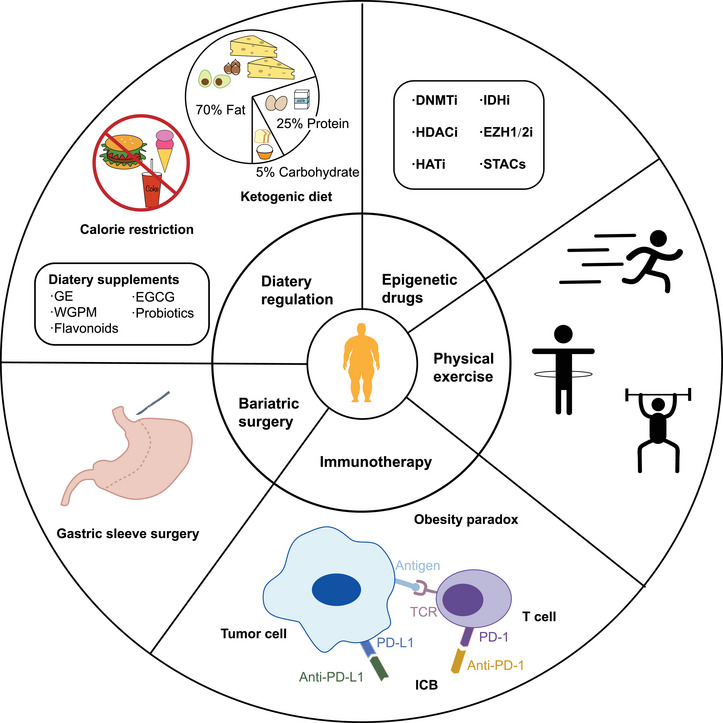
Prevention and therapy in obesity‐associated diseases. Weight loss has long been a topic of intense interest and research, leading to the emergence of various approaches and methods over the years. From traditional calorie restriction and exercise to newer strategies like the ketogenic diet and bariatric surgery, the landscape of weight loss interventions continues to evolve. Currently, the predominant approach to weight loss involves regular physical exercise in conjunction with dietary regulation. Calorie restriction can lead to weight loss through negative nitrogen balance, but excessive dieting may cause pronounced hunger and reduce basal metabolism. Moderate calorie restriction combined with exercise can elevate the basal metabolic rate, establish a new balance between energy intake and expenditure, and maintain a healthy physical state. The ketogenic diet has emerged as a novel approach to weight loss in recent years, typically characterized by a diet ratio of 70% fat, 25% protein, and 5% carbohydrate. The ketogenic diet simulates the body's fasting state, promotes fat mobilization, reduces blood insulin levels, and can lead to weight loss in the short term. Nonetheless, prolonged application of the ketogenic diet is not recommended due to the potential for generating excessive ketone bodies, which can induce electrolyte disorder and increase burden on kidney. Furthermore, research indicates that adding certain natural compounds and probiotics into the diet may reduce the risk of obesity complications. Bariatric surgery is primarily indicated for individuals with significant obesity who struggle to achieve weight loss through conventional methods such as exercise and dieting. Bariatric surgery has also demonstrated the ability to counteract the adverse effects of obesity. Intriguingly, some obese cancer patients exhibit heightened sensitivity to ICB treatment, which can help further optimize ICB treatment strategies. The advent of epigenetic drugs also presents new therapeutic approaches for addressing obesity‐associated diseases. Combining highly targeted epigenetic drugs with conventional treatment regimens can enhance therapeutic effectiveness and mitigate side effects. GE, genistein: WGPM, whole grain proso millet; EGCG, epigallocatechin‐3‐gallate; DNMTi, inhibitor of DNA methyltransferases; HDACi, inhibitor of histone deacetylases; IDHi, Inhibitor of the mutant isocitrate dehydrogenase enzymes; EZH1/2i, inhibitor of enhancer of zeste homolog 1/2; STACs, sirtuin‐activating compounds; TCR, T cell receptor; ICB, immune checkpoint blockade; PD‐1, programmed cell death protein 1; PD‐L1, programmed death‐ligand 1.

Obesity is a significant contributing factor to various metabolic disorders and certain types of cancer. Reversing obesity state represents a potential breakthrough in the prevention and treatment of these conditions. Managing weight presents a significant challenge for obese individuals, and achieving successful weight control necessitates a combination of diverse approaches and long‐term persistence. Dietary regulation, physical exercise, and bariatric surgery can reduce the risk of obesity‐associated diseases by addressing weight control at the root, while epigenetic drugs and immunotherapy can enhance the effectiveness of conventional treatments for obesity‐associated diseases (Figure 4). According to classical genetic theory, phenotypic changes are based on changes in DNA sequences. However, with the emergence of epigenetics, mechanisms that induce phenotypic alterations are becoming varied and complex. Exploring the epigenetic modifications of diseases is helpful to the prevention and treatment. In particular, specific epigenetic changes could be considered valuable biological markers in the predisease state and early stage of diseases development, which can provide new methods to detect and treat diseases early and prolong the lifespan of patients. Hence, exploring the field of epigenetic modification is vital as it provides prospects for clinical application.

### 7.1 Dietary regulation

With the change in dietary habits, high‐fat/fast food has become a common dish on the table. Consumption of a HFD can alter the global DNAm patterns, thereby elevating the susceptibility to obesity‐associated diseases.^232^, ^233^ Rats fed a high‐sugar and high‐fat cafeteria diet exhibited adipocyte hypertrophy and IR in the VAT, which was associated with DNA hypomethylation of the solute carrier family 27 member 3 (Slc27a3).^234^ An obese diet could also affect the gut microbiome to stimulate histone modification of enhancers in the colon, which might be relative to the promotion of colon cancer.^104^ Moreover, HFD, which is rich in dietary vegetable fat n‐6 linoleic acid, was known to regulate DNAm alterations of farnesoid‐X‐receptor (*Fxr*) and prostaglandin‐endoperoxide synthase‐2 (*Ptsg‐2*) genes, which involve in the development of colonic inflammation and cancer.^235^ Over‐consumption of fructose for an extended period might promote lipid accumulation and downregulated expression of LEP and miR‐192, which was also related to prostate cancer pathogenesis.^236^ Excessive fructose consumption elevates the risk of developing T2D by impacting the regulatory network of miRNAs, thereby diminishing insulin sensitivity and promoting the accumulation of TG.^237^ Consequently, it is crucial to restrict fructose consumption in our regular diet. Proper dietary interventions can mitigate the adverse effects of obesity and decrease the initiation and progress of associated complications.^232^ Research demonstrated that following a two‐year weight loss dietary intervention, TXNIP DNA hypermethylation, regulating glucose homeostasis, enhanced pancreatic islet function and lowered the risk of T2D.^238^ In addition, dietary intervention for individuals in early T2D has the potential to mitigate the impairment on β cells due to lipotoxicity and inflammation by reinstating the chromatin reprogramming.^210^ Improved diet quality can alter the levels of DNAm in cardiometabolism‐related genes, which is linked with increased risk of hypertension and CHDs.^239^ Energy restriction has always been the main intervention for weight loss, but it poses significant challenges to long‐term dietary intervention due to its impact on health and low compliance.

Certain nutrients are thought to mitigate the impact of obesity, and their proper intake may help prevent obesity‐associated diseases. Animal research suggested that adding grape powder to HFD could reduce oncomiR‐34a and oncomiR‐21 expression to prevent obesity‐induced prostate cancer.^240^ Genistein (GE) is a bioactive nutritional compound that is abundant in soybean products and is considered a potential antitumor agent. Nowadays, GE consumption could prevent advanced BC through epigenetic modification and even inhibit HFD‐induced BC promotion and metabolism disorders in offspring.^241^ Maternal consumption of GE can impact the initial colonization of intestinal microbiota, global DNAm levels and the expression of key cancer‐associated genes in offspring.^242^ Whole grain proso millet (WGPM) has been shown to substantially reduce blood glucose and lipid levels in mice with T2D by altering the expression profile of miRNA and inhibiting gluconeogenesis‐related pathways.^243^ Flavonoids are prevalent in plants and have demonstrated the ability to enhance endothelial function and decrease the CVDs risk through epigenetic regulation.^244^ Epigallocatechin‐3‐gallate (EGCG), which is rich in green tea, can act as an epigenetic regulator to inhibit ROS production and exert an antioxidant effect, contributing to prevent CVDs and diabetes.^245^ The gut microbiota in obese patients diminishes ethanolamine metabolism, elevates intestinal permeability, and contributes to chronic inflammation. Innovative probiotics have the potential to reinstate microbial ethanolamine metabolism and alleviate intestinal permeability and inflammation in obese and diabetic patients by targeting the AT‐rich interaction domain 3a (ARID3a)/miR‐101a/zona occludens‐1 (Zo1) axis.^246^ Furthermore, supplementing the diet with new probiotics can decrease the levels of miR‐155‐5p and miR‐125b‐5p in the plasma of obese patients, thereby regulating inflammation and lipogenesis.^247^ While dietary supplements are generally a safe and convenient means of prevention, additional clinical experimental evidence is needed to determine appropriate optimal dosages and supplementation method.

When glucose levels are insufficient under the state of starvation, the lipids are decomposed into glycerol and fatty acids by fat mobilization, producing acetyl‐CoA for energy and ketone bodies to supply the brain and heart. The ketogenic diet is characterized by high‐fat and low‐carbohydrate, similar to the state of hunger, producing a large number of ketone bodies. At present, studies have shown that a ketogenic diet helps to inhibit the progression of metabolic syndrome, diabetes, CVDs, and cancer.^248^, ^249^, ^250^ Nevertheless, some studies indicate that a long‐term ketogenic diet may lead to IR and elevated LDL levels.^251^, ^252^ Consequently, numerous uncertainties persist regarding the effectiveness of ketogenic diet treatment for metabolic diseases. We all know that cancer cells generate energy mainly through glycolysis, and damaged mitochondrial function prevents them from utilizing ketone bodies for energy production like normal cells. Thus, tumor cells are more sensitive to glucose deficiency. A ketogenic diet inhibits the initiation of the primary tumor and systemic metastasis by lower blood glucose levels and increasing ketone bodies. However, the biological mechanisms of the antitumor effect of ketone bodies remain unclear. The latest study showed that β‐hydroxybutyric acid, a ketone body, could bind to the cell surface receptor Hcar2, increasing the level of transcription factor Hopx to suppress the proliferation of colon cancer cells.^253^ β‐hydroxybutyric acid can mediate histone lysine 3‐hydroxybutyrylation modification which has been identified as a noval epigenetic modification that links fatty acid metabolism to cancer progession.^254^ β‐Hydroxybutyrate dehydrogenase 1 (BDH1) is the primary rate‐limiting enzyme in ketone body metabolism and is closely related to the overall survival rate of patients with HCC. Metastasis‐associated protein 2 (MTA2) could inhibit the level of BDH1 and increase histone β‐hydroxybutyrylation, thus promoting the proliferation of HCC stem cells.^255^ β‐Hydroxybutyrylation of tumor suppressor factor p53 could inhibit cancer cell apoptosis and dampen the function that maintains cancer cell growth arrest.^256^ However, β‐hydroxybutyrylation might also play an antitumor role in inhibiting the critical enzyme of methionine metabolism, as tumors are highly dependent on methionine metabolism.^257^ There are diverse results treated with ketone bodies in different tumors under different physiological conditions. While the ketogenic diet may impede the tumor's growth rate, persistent weight loss may engender cachexia. Recent studies indicate that the utilization of glucocorticoids alongside a ketogenic diet may avert the onset of cachexia and correspondingly elongate survival rates.^258^ To date, most of the experiments conducted have only been based on animal models, and further studies are required to determine how the ketogenic diet can be safely applied in clinical anticancer therapy.

### 7.2 Physical exercise

In addition to healthy dietary intervention, regular physical exercise is also an effective preventive measure for obesity‐associated diseases.^259^, ^260^, ^261^, ^262^ In a study focusing on postmenopausal overweight/obese women, miR‐122 levels in the plasma of the participants decreased significantly following a 12‐month weight‐loss regimen of diet and exercise that reduced BC risk.^263^ Physical exercise can stimulate the release of ncRNAs and alleviate metabolic abnormalities by impacting intertissue communication.^264^ The myokine irisin, primarily derived from contracted skeletal muscles, plays a key role in improving glucose tolerance and suppressing IR.^265^ From a mechanistic standpoint, miRNAs released from skeletal muscle may be involved in the regulation of irisin expression.^265^ Exercise can influence the miRNA expression profile in exosomes derived from myotubes and upregulate the expression of genes associated with anti‐inflammatory responses and appetite regulation.^266^ The effects of exercise are comprehensive, and designing a tailored exercise plan based on individual conditions can enhance insulin sensitivity and cardiopulmonary health. Integrating it with dietary regulation can produce multiplied benefits.

### 7.3 Bariatric surgery

A sedentary state is ubiquitous during daily study and work, and physical exercise and calorie restriction are two major measures to manage weight. Although caloric restriction can limit the glucose supply to tumors, it can perturb water and electrolyte metabolism, causing physical damage to patients. Therefore, it is not advisable for patients to attempt this method without professional guidance. Furthermore, recent research suggested that long‐term weight loss interventions had a better effect on cancer prevention and mitigation than short‐term approaches.^20^, ^267^, ^268^ Epigenetic modification is a dynamic process that can be reversed by long‐term weight loss interventions, thereby reducing the risk of obesity‐associated diseases. The potential harmful effects of obesity are well‐documented. In young individuals, obesity can lead to changes in DNAm, and over time, tumor‐prone gene signature appears in aging individuals.^269^ Obesity‐associated DNAm changes and the upregulation of oncogenes remain unchanged after short‐term weight loss, but long‐term weight loss reverses the tumor‐prone gene signature.

Except for diet control and physical exercise, long‐term weight loss induced by bariatric surgery could change the metabolic status through epigenetic regulation, thus reducing the risk of obesity‐related complications.^270^, ^271^ According to a cohort study of 30318 obese patients (BMI ≥ 35) that showed bariatric surgery significantly reduced the incidence and mortality of obesity‐associated cancers.^272^ The plasma miRNA expression profile in T2D patients undergoes changes following bariatric surgery. This analysis of changes can help identify potential therapeutic targets for T2D. Differentially expressed miRNAs in obese patients tended to be normalized after bariatric surgery, which was thought to reduce the risk of colorectal cancer.^273^ Gastric sleeve surgery can prompt the upregulation of tumor suppressor miR‐122 in serum, thus reducing the risk of cancer.^274^ In addition, the marked upregulation of lncRNA Gm19619 triggered by a HFD can enhance hepatic gluconeogenesis and lipid accumulation.^275^ Following vertical sleeve gastrectomy, the expression of lncRNA Gm19619 is reduced, leading to improved glucose and lipid metabolism.^275^ Sleeve gastrectomy can reverse the upregulation of lncRNA taurine‐upregulated gene 1 (TUG1) induced by high sugar and high fat intake, leading to decreased TUG1 levels which in turn help in regulating blood sugar levels in patients with T2D.^276^ Hence, bariatric surgery presents a promising approach for preventing complications in obese patients.

### 7.4 Epigenetic drugs

The diversity and uncertainty of epigenetic modification pose significant challenges to drug therapy related to epigenetics. Resveratrol or 5‐aza could reduce triglyceride accumulation in hepatoma cells, and the resveratrol could reverse promoter DNAm of Nrf2‐Keap1 (aberrant DNAm in human cancers) to attenuate NAFLD.^277^ HCC patients induced by NAFLD exhibit overexpression of Squalene Epoxidase (SQLE), which triggers a series of oxidative stress events. These events include the expression of DNMT3A and the silencing of DNMT3A‐induced Phosphatase and Tensin Homolog (PTEN), ultimately activating AKT–mTOR.^278^ Inhibitors of SQLE, such as the antifungal drug terbinafine, could suppress this series of chain reactions and offer a new approach for HCC target therapy. As a target point, the fatty acid‐binding (FABP4) could indirectly lead to overexpression of DNMT1 and silence tumor suppressor gene p15^INK4B^ in AML cells.^279^ Bioactive compound honokiol and adiponectin could inhibit leptin‐induced tamoxifen resistance in BC.^153^ Maternal generation with HFD during pregnancy can induce epigenetic alteration in offspring. DNMT inhibitor hydralazine and HDAC inhibitor valproic acid suppresses BC progress in mice offspring of the high‐fat group but increases the incidence and burden of BC in the control group.^280^ Currently, approved epigenetic drugs are mainly prescribed for treating hematological malignant tumors (Table 1), while a large number of epigenetic drugs for solid tumors are under clinical trials. This will provide a new direction for cancer treatment.

**TABLE 1:** Epigenetic drugs for hematologic malignancies in clinic application.

Type of drug	Mechanism of drug	Drug name	Cancer type
Inhibitor of DNA methyltransferases (DNMTi)	Demethylation under low concentrations and cytotoxic effects under high concentrations	Azacitidine^291^	Myelodysplastic syndromes, MDS
		Decitabine^292^	
Inhibitor of enhancer of zeste homolog 1/2 (EZH1/2i)	Inhibiting histone methyltransferase enhancer of zeste homolog 1/2 (EZH1/2i) activity to regulate the expression level of cancer‐related genes, and impede abnormal cell proliferation	Tazemetostat^293^ Valemetostat^294^	Epithelioid Sarcoma, ES Relapsed or refractory adult T‐cell leukemia/lymphoma, R/R ATL
Inhibitor of histone deacetylases (HDACi)	Increasing the transcription of genes associated with tumor suppression, cell cycle stagnation, cell differentiation, and apoptosis under the slack chromatin state	Vorinostat^295^	Cutaneous T cell lymphoma, CTCL
		Belinostat^296^	Peripheral T cell lymphoma, PTCL
		Romidepsin^297^	CTCL and PTCL
		Chidamide^298^	PTCL
		Panobinostat^299^	Multiple Myeloma, MM
Inhibitor of the mutant isocitrate dehydrogenase enzymes (IDHi)	Combined with mutant IDH1/IDH2 can reduce the level of tumor metabolite 2‐hydroxyglutarate (2‐HG) and promote normal cell differentiation	Ivosidenib^300^	IDH1‐mutated AML
		Enasidenib^301^	IDH2‐mutated AML

Though there are no epigenetic drugs approved for the treatment of T2D and CVDs in clinical application, some drugs are undergoing clinical trials (Table 2). Investigating the epigenetic genome of obese patients and advancing the development of targeted chromatin‐modifying drugs have the potential to minimize side effects and facilitate precision treatment.^86^ Thiazolidinediones are antidiabetic drugs that target PPARγ, but their usage is restricted due to their potential impact on heart and kidney function. HDAC3 inhibitors have the ability to induce posttranslational modifications of PPARγ and could potentially offer a safer therapeutic approach for T2D by enhancing insulin sensitivity.^107^ Bromodomain and extraterminal (BET) proteins act as histone acetylation readers in chromatin remodeling. The BET inhibitor apabetalone has the potential to reduce the risk of CVDs and T2D by suppressing monocyte inflammatory activation.^281^

**TABLE 2:** Epigenetic drugs for therapy of diabetes and CVDs in the clinic trail.

Type of drug	Drug name	Diseases type	Phase	NCT number
DNMTi	Hydralazine	T2D/atherosclerosis/CVDs/ hypercholesterolemia/ hypertension	Phase III	NCT00000620
		Diabetic nephropathy	Phase IV	NCT02046395
		Hypertension	Phase II	NCT00000499
		Hypertensive renal disease	Phase II/III	NCT00582777
		Heart Failure	Phase II/III	NCT01255475
			Phase II	NCT01516346
HDACi	Sodium phenylbutyrate	Diabetes/IR	Phase IV	NCT00533559
			Not applicable	NCT00771901
		Prediabetics	Not applicable	NCT05028803
	Ricolinostat	Painful diabetic peripheral neuropathy	Phase II	NCT03176472
Inhibitor of histone acetyltransferases (HATi)	Curcumin	T2D	Phase IV	NCT04528212
		T2D (IR and oxidative stress)	Not applicable	NCT02529982
		Metabolic syndrome (gut barrier function)	Not Applicable	NCT03542240
		NAFLD with T2D	Phase II/III	NCT02908152
		Hypercholesterolemia/ hypertension	Not applicable	NCT03542240
		Atherosclerosis	Not applicable	NCT02998918
Sirtuin‐activating compounds (STACs)	Resveratrol	T2D (endothelial function)	Not applicable	NCT01038089
			Not applicable	NCT01881347
		T2D	Phase I	NCT01677611
		T2D (inflammation)	PhaseIII	NCT02244879
		Diabetes with neuropathic complication	Not applicable	NCT05172947
		Prediabetics	Not applicable	NCT02565979
		Metabolic syndrome	Not applicable	NCT01714102
		Diabetic nephropathy	Early Phase I	NCT02704494
		Heart failure (metabolic and skeletal muscle function)	Phase II	NCT03525379
		Diastolic heart failure/ hypertension	Phase I	NCT01185067
		Coronary artery disease	Not applicable	NCT02137421
			Not applicable	NCT06095635
		CVDs (postmenopausal women)	Phase I/II	NCT01564381
		CVDs (primary and secondary Prevention)	Phase II	NCT01449110
		Atherosclerosis	Not applicable	NCT02998918
		Peripheral artery disease	Phase III	NCT03743636
			Not applicable	NCT02246660
		Vascular system injuries/endothelial disfunction	Not applicable	NCT01668836
		Hypertension	Phase I	NCT02616822
		Hypercholesterolemia	Phase II	NCT02409537

*Data resource*: https://www.clinicaltrials.gov.

In comparison with traditional drugs, epigenetic drugs show certain advantages, the development of epigenetic drugs faces significant challenges due to high costs and individual variability. Although epigenetic therapy has brought great convenience to diseases treatment, it still has a long way to go before its wide application in clinics.

### 7.5 Immunotherapy in obesity‐associated cancers

Tumor cells evade immune surveillance and malignantly proliferate in the organism due to their low immunogenicity and the release of immune suppressing molecules. Recent years, immunotherapy targeting tumor immune evasion has brought hope to cancer patients. However, the response to immunotherapy varies among patients with obesity‐associated cancers, and the specific mechanisms by which obesity affects the tumor immune microenvironment are not clear. In general, with the progression of obesity, the infiltration of myeloid‐derived suppressor cells increases in tumors, while CD8+ T cells decrease, leading to inhibition of antitumor immunity.^282^, ^283^ However, some obese patients show better response to immunotherapy compared with normal‐weight patients. For instance, researchers have constructed a DNAm‐based biomarker for BMI (DM‐BMI) model to investigate the relationship between obesity and BC. They found a positive correlation between DM‐BMI and immunotherapy checkpoint inhibitor response markers, indicating that obese cancer patients may be more sensitive to immune checkpoint blockade therapy than normal‐weight patients.^284^ In obese patients, increased interferon‐gamma and leptin can upregulate the expression of PD‐1/PD‐L1, and anti‐CTLA‐4 therapy has also been demonstrated to be more effective.^285^ In addition, the expression of Nod‐like receptor family pyrin domain containing 3 (NLRP3) inflammasome increases in AT of obese patients.^286^ The NLRP3 inflammasome can produce inflammatory factors, including IL‐1β and IL‐18, while promoting the upregulation of PD‐1/PD‐L1 expression, resulting in an immune‐suppressive microenvironment.^286^ Current research indicates that miR‐223‐3p can downregulate the NLRP3 inflammasome, thereby mitigating tumor progression and offering a novel approach to overcoming immunotherapy resistance.^287^ Variances in obese cancer patients’ responsiveness to immunotherapy may be associated with obesity‐induced epigenetic modifications. Subsequent research may unveil novel therapeutic targets for cancer. Presently, the confluence of immunotherapy and chemotherapy with epigenetic drug therapy has the potential to augment the efficacy of singular treatments while mitigating adverse drug effects, thus emerging as promising anticancer strategies.

## 8 CONCLUSION

The increased prevalence of obesity can be attributed to the daily consumption of high‐energy diets and a reduction in physical activity levels. Except for stirring up physical anxiety, obesity is considered to weaken immunity system, lead to metabolic and endocrine dysfunction, disrupt fertility, and its long‐term chronic inflammation exacerbates obesity complications incidence and progression. Obesity alters the overall metabolic environment, and adverse signals from AT can be relayed to adjacent or distant organs through intercellular communication. AT is one of the earliest sites of IR to appear. In obesity, AT can lead to aberrant expression of adiponectin, leptin, and other adipokines through epigenetic modifications. This can impact β cell insulin secretion, induce inflammatory responses, trigger endothelial dysfunction, myocardial damage, and contribute to cardiometabolic diseases. Additionally, adipokines can regulate angiogenesis, cell proliferation and reshape TME to promote cancer development. Excessive fat accumulation can induce macrophage infiltration in AT, and increase the release of inflammation factors through epigenetic regulation, aggravating the level of inflammation.

Currently, obesity is a significant risk factor for cancer, T2D and CVDs. Interventions for AT‐induced inflammatory state, and lifestyle changes such as diet, exercise, and even surgery can reverse the obesity‐induced abnormal signaling pathways. The temporary and reversible nature of epigenetics provide a noval treatment option for obesity‐associated diseases. The emergence of epigenetic editing has offered technical support for investigating disease pathogenesis and clinical translation.^288^ However, epigenetic editing technology is associated with drawbacks such as off‐target effects and limited short‐term efficacy, while delivery of CRISPR/Cas in vivo encounters substantial challenges.^289^ In addition, with the appearance of genetic and epigenetic landscape and entropy, we have a more comprehensive understanding of diseases pathogenesis, and more efficient targeted drugs were invented with new experimental approaches.^290^ To date, a variety of epigenetic drugs are in the onset stage. Epigenetic drugs have the capability to precisely target specific genes and signaling pathways, enabling tailored treatment. Moreover, their reversibility allows for dynamic treatment strategies. Epigenetic drugs hold promise for targeting diverse diseases and have broad applicability. Nevertheless, the off‐target effects of epigenetic drugs may lead to unforeseen side effects by targeting unintended genes or pathways, and the potential long‐term impact on the body remains unconfirmed. Consequently, achieving high selectivity and ensuring safety are critical challenges in the development of epigenetic drugs.

Meanwhile, detection of DNAm and ncRNAs offer a noninvasive means of diagnosing obesity‐associated diseases, providing convenience to patients. The emergence of exosomes also facilitates the application of exogenous small ncRNAs for therapy. Currently, exogenous ncRNAs are mainly transported by adipocytes‐derived exosomes, which makes target positioning difficult and may cause negative effects on nontargeted cells or organs. The high cost of large‐scale production and unknown safety have hindered the clinical application of ncRNAs. In the future, people should overcome the difficulty of delivering different ncRNAs at the same time and focus on developing safer and more specific vehicles. In summary, epigenetics holds significant therapeutic potential for obesity‐associated diseases, yet it is confronted with ethical, safety, and feasibility challenges, necessitating continual improvement and validation in clinical practice.

## AUTHOR CONTRIBUTIONS

*Writing—original draft; conceptualization; visualization*: Yiqian Long. *Supervision; funding acquisition*: Chao Mao. *Project administration; funding acquisition*: Shuang Liu. *Writing—review and editing; project administration; supervision; funding acquisition*: Yongguang Tao. *Writing—review and editing; project administration; supervision; funding acquisition*: Desheng Xiao.

All authors have read and approved the article.

## CONFLICT OF INTEREST STATEMENT

All authors disclosed no relevant relationships.

## ETHICS STATEMENT

Not applicable.

## Data Availability

Not applicable.

## References

[1] Obesity and overweight. Updated 9 June 2021. Accessed 10 January, 2024. https://www.who.int/news‐room/fact‐sheets/detail/obesity‐and‐overweight

[2] Oliveros E , Somers VK , Sochor O , Goel K , Lopez‐Jimenez F . The concept of normal weight obesity. Prog Cardiovasc Dis. 2014;56(4):426‐433. 24438734 10.1016/j.pcad.2013.10.003

[3] Pluta W , Dudzińska W , Lubkowska A . Metabolic obesity in people with normal body weight (MONW)‐review of diagnostic criteria. Int J Environ Res Public Health. 2022;19(2). 10.3390/ijerph19020624 PMC8776153 35055447

[4] Sung H , Siegel RL , Torre LA , et al. Global patterns in excess body weight and the associated cancer burden. CA Cancer J Clin. 2019;69(2):88‐112. 30548482 10.3322/caac.21499

[5] Arnold M , Leitzmann M , Freisling H , et al. Obesity and cancer: an update of the global impact. Cancer Epidemiol. 2016;41:8‐15. 26775081 10.1016/j.canep.2016.01.003

[6] Sun M , Fritz J , Häggström C , et al. Metabolically (un)healthy obesity and risk of obesity‐related cancers: a pooled study. J Natl Cancer Inst. 2023;115(4):456‐467. 36647199 10.1093/jnci/djad008 PMC10086630

[7] Ligibel JA , Alfano CM , Courneya KS , et al. American Society of Clinical Oncology position statement on obesity and cancer. J Clin Oncol. 2014;32(31):3568‐3574. 25273035 10.1200/JCO.2014.58.4680 PMC4979237

[8] Siegel RL , Miller KD , Wagle NS , Jemal A . Cancer statistics, 2023. CA Cancer J Clin. 2023;73(1):17‐48. 36633525 10.3322/caac.21763

[9] Apovian CM , Okemah J , O'Neil PM . Body weight considerations in the management of type 2 diabetes. Adv Ther. 2019;36(1):44‐58. 30465123 10.1007/s12325-018-0824-8 PMC6318231

[10] Kahn SE , Cooper ME , Del Prato S . Pathophysiology and treatment of type 2 diabetes: perspectives on the past, present, and future. Lancet. 2014;383(9922):1068‐1083. 24315620 10.1016/S0140-6736(13)62154-6 PMC4226760

[11] Liu Y , Douglas PS , Lip GYH , et al. Relationship between obesity severity, metabolic status and cardiovascular disease in obese adults. Eur J Clin Invest. 2023;53(3):e13912. 36424669 10.1111/eci.13912

[12] Powell‐Wiley TM , Poirier P , Burke LE , et al. Obesity and cardiovascular disease: a scientific statement from the American Heart Association. Circulation. 2021;143(21):e984‐e1010. 33882682 10.1161/CIR.0000000000000973 PMC8493650

[13] Jin X , Qiu T , Li L , et al. Pathophysiology of obesity and its associated diseases. Acta Pharm Sin B. 2023;13(6):2403‐2424. 37425065 10.1016/j.apsb.2023.01.012 PMC10326265

[14] Liu L , Shi Z , Ji X , et al. Adipokines, adiposity, and atherosclerosis. Cell Mol Life Sci. 2022;79(5):272. 35503385 10.1007/s00018-022-04286-2 PMC11073100

[15] Rojas E , Rodríguez‐Molina D , Bolli P , et al. The role of adiponectin in endothelial dysfunction and hypertension. Curr Hypertens Rep. 2014;16(8):463. 24924994 10.1007/s11906-014-0463-7

[16] Ruze R , Liu T , Zou X , et al. Obesity and type 2 diabetes mellitus: connections in epidemiology, pathogenesis, and treatments. Front Endocrinol (Lausanne). 2023;14:1161521. 37152942 10.3389/fendo.2023.1161521 PMC10161731

[17] Kim DS , Scherer PE . Obesity, diabetes, and increased cancer progression. Diabetes Metab J. 2021;45(6):799‐812. 34847640 10.4093/dmj.2021.0077 PMC8640143

[18] Hill MA , Yang Y , Zhang L , et al. Insulin resistance, cardiovascular stiffening and cardiovascular disease. Metabolism. 2021;119:154766. 33766485 10.1016/j.metabol.2021.154766

[19] Cavalli G , Heard E . Advances in epigenetics link genetics to the environment and disease. Nature. 2019;571(7766):489‐499. 31341302 10.1038/s41586-019-1411-0

[20] Li R , Grimm SA , Mav D , et al. Transcriptome and DNA methylome analysis in a mouse model of diet‐induced obesity predicts increased risk of colorectal cancer. Cell Rep. 2018;22(3):624‐637. 29346762 10.1016/j.celrep.2017.12.071 PMC5793878

[21] Izquierdo‐Torres E , Hernández‐Oliveras A , Lozano‐Arriaga D , Zarain‐Herzberg Á . Obesity, the other pandemic: linking diet and carcinogenesis by epigenetic mechanisms. J Nutr Biochem. 2022;108:109092. 35718098 10.1016/j.jnutbio.2022.109092

[22] Ling C , Rönn T . Epigenetics in human obesity and type 2 diabetes. Cell Metab. 2019;29(5):1028‐1044. 30982733 10.1016/j.cmet.2019.03.009 PMC6509280

[23] Jurkowska RZ , Jurkowski TP , Jeltsch A . Structure and function of mammalian DNA methyltransferases. Chembiochem. 2011;12(2):206‐222. 21243710 10.1002/cbic.201000195

[24] Chen Z , Zhang Y . Role of mammalian DNA methyltransferases in development. Annu Rev Biochem. 2020;89:135‐158. 31815535 10.1146/annurev-biochem-103019-102815

[25] Jones PA . Functions of DNA methylation: islands, start sites, gene bodies and beyond. Nat Rev Genet. 2012;13(7):484‐492. 22641018 10.1038/nrg3230

[26] Wu X , Zhang Y . TET‐mediated active DNA demethylation: mechanism, function and beyond. Nat Rev Genet. 2017;18(9):517‐534. 28555658 10.1038/nrg.2017.33

[27] Bochtler M , Kolano A , Xu GL . DNA demethylation pathways: additional players and regulators. Bioessays. 2017;39(1):1‐13. 10.1002/bies.201600178 27859411

[28] Jones PA , Baylin SB . The epigenomics of cancer. Cell. 2007;128(4):683‐692. 17320506 10.1016/j.cell.2007.01.029 PMC3894624

[29] Huang T , Huang X , Nie Y , Shi X , Shu C . A combined effect of expression levels of obesity‐related genes and clinical factors on cancer survival rate. BioMed Res Int. 2020;2020:8838676. 33299884 10.1155/2020/8838676 PMC7707943

[30] Torres‐Gutierrez CJ , Heckman MG , Hlady RA , et al. Association of clinical epidemiologic exposures and overall survival with genome‐wide DNA methylation profiles in acute myeloid leukemia: analysis of the mayo clinic AML epidemiology cohort. Blood. 2018;132:3987.

[31] Crujeiras AB , Morcillo S , Diaz‐Lagares A , et al. Identification of an episignature of human colorectal cancer associated with obesity by genome‐wide DNA methylation analysis. Int J Obes (Lond). 2019;43(1):176‐188. 29717273 10.1038/s41366-018-0065-6

[32] Crujeiras AB , Diaz‐Lagares A , Stefansson OA , et al. Obesity and menopause modify the epigenomic profile of breast cancer. Endocr Relat Cancer. 2017;24(7):351‐363. 28442560 10.1530/ERC-16-0565

[33] Lorenzo PM , Izquierdo AG , Diaz‐Lagares A , et al. ZNF577 methylation levels in leukocytes from women with breast cancer is modulated by adiposity, menopausal state, and the mediterranean diet. Front Endocrinol. 2020;11:245. 10.3389/fendo.2020.00245 PMC7191069 32390948

[34] Cabrera‐Mulero A , Crujeiras AB , Izquierdo AG , et al. Novel SFRP2 DNA methylation profile following neoadjuvant therapy in colorectal cancer patients with different grades of BMI. J Clin Med. 2019;8(7):1041. 10.3390/jcm8071041 PMC6678889 31319558

[35] Castellano‐Castillo D , Morcillo S , Crujeiras AB , et al. Association between serum 25‐hydroxyvitamin D and global DNA methylation in visceral adipose tissue from colorectal cancer patients. BMC Cancer. 2019;19(1):93. 10.1186/s12885-018-5226-4 PMC6341579 30665376

[36] Frederick ALM , Guo C , Meyer A , Yan L , Schneider SS , Liu Z . The influence of obesity on folate status, DNA methylation and cancer‐related gene expression in normal breast tissues from premenopausal women. Epigenetics. 2021;16(4):458‐467. 32749195 10.1080/15592294.2020.1805687 PMC7993144

[37] Bao B , Teslow EA , Mitrea C , Boerner JL , Dyson G , Bollig‐Fischer A . Role of TET1 and 5hmC in an obesity‐linked pathway driving cancer stem cells in triple‐negative breast cancer. Mol Cancer Res. 2020;18(12):1803‐1814. 32913111 10.1158/1541-7786.MCR-20-0359 PMC7718329

[38] Dong L , Ma L , Ma GH , Ren H . Genome‐wide analysis reveals DNA methylation alterations in obesity associated with high risk of colorectal cancer. Sci Rep. 2019;9(1):5100. 30911103 10.1038/s41598-019-41616-0 PMC6433909

[39] Rodríguez‐Miguel C , Moral R , Escrich R , Vela E , Solanas M , Escrich E . The role of dietary extra virgin olive oil and corn oil on the alteration of epigenetic patterns in the rat DMBA‐induced breast cancer model. PLoS One. 2015;10(9):e0138980. 26401660 10.1371/journal.pone.0138980 PMC4581736

[40] Sedaghat F , Cheraghpour M , Hosseini SA , et al. Hypomethylation of NANOG promoter in colonic mucosal cells of obese patients: a possible role of NF‐κB. Br J Nutr. 2019;122(5):499‐508. 30157990 10.1017/S000711451800212X

[41] Milner JJ , Chen ZF , Grayson J , Shiao SPK . Obesity‐associated differentially methylated regions in colon cancer. J Pers Med. 2022;12(5):660. 10.3390/jpm12050660 PMC9142939 35629083

[42] Tian Y , Arai E , Makiuchi S , et al. Aberrant DNA methylation results in altered gene expression in non‐alcoholic steatohepatitis‐related hepatocellular carcinomas. J Cancer Res Clin Oncol. 2020;146(10):2461‐2477. 32685988 10.1007/s00432-020-03298-4 PMC7467955

[43] Cheng Y , Monteiro C , Matos A , et al. Epigenome‐wide DNA methylation profiling of periprostatic adipose tissue in prostate cancer patients with excess adiposity‐a pilot study. Clin Epigenet. 2018;10(1):54. 10.1186/s13148-018-0490-3 PMC5904983 29692867

[44] Boughanem H , Ruiz‐Limon P , Crujeiras AB , de Luque V , Tinahones FJ , Macias‐Gonzalez M . 25‐Hydroxyvitamin D status is associated with interleukin‐6 methylation in adipose tissue from patients with colorectal cancer. Food Funct. 2021;12(20):9620‐9631. 34549226 10.1039/d1fo01371h

[45] Spyrou N , Avgerinos KI , Mantzoros CS , Dalamaga M . Classic and novel adipocytokines at the intersection of obesity and cancer: diagnostic and therapeutic strategies. Curr Obes Rep. 2018;7(4):260‐275. 30145771 10.1007/s13679-018-0318-7

[46] Herrera‐Vargas AK , García‐Rodríguez E , Olea‐Flores M , Mendoza‐Catalán MA , Flores‐Alfaro E , Navarro‐Tito N . Pro‐angiogenic activity and vasculogenic mimicry in the tumor microenvironment by leptin in cancer. Cytokine Growth Factor Rev. 2021;62:23‐41. 34736827 10.1016/j.cytogfr.2021.10.006

[47] Hsieh YY , Shen CH , Huang WS , et al. Resistin‐induced stromal cell‐derived factor‐1 expression through Toll‐like receptor 4 and activation of p38 MAPK/NFκB signaling pathway in gastric cancer cells. J Biomed Sci. 2014;21(1):59. 24929539 10.1186/1423-0127-21-59 PMC4089564

[48] Shi Y , Zhu N , Qiu Y , et al. Resistin‐like molecules: a marker, mediator and therapeutic target for multiple diseases. Cell Commun Signal. 2023;21(1):18. 36691020 10.1186/s12964-022-01032-w PMC9869618

[49] Polusani SR , Huang YW , Huang G , et al. Adipokines deregulate cellular communication via epigenetic repression of gap junction loci in obese endometrial cancer. Cancer Research. 2019;79(1):196‐208. 30389702 10.1158/0008-5472.CAN-18-1615 PMC6705596

[50] Bokobza E , Hinault C , Tiroille V , Clavel S , Bost F , Chevalier N . The adipose tissue at the crosstalk between EDCs and cancer development. Front Endocrinol (Lausanne). 2021;12:691658. 34354670 10.3389/fendo.2021.691658 PMC8329539

[51] Basak S , Das MK , Duttaroy AK . Plastics derived endocrine‐disrupting compounds and their effects on early development. Birth Defects Res. 2020;112(17):1308‐1325. 32476245 10.1002/bdr2.1741

[52] Kahn LG , Philippat C , Nakayama SF , Slama R , Trasande L . Endocrine‐disrupting chemicals: implications for human health. Lancet Diabetes Endocrinol. 2020;8(8):703‐718. 32707118 10.1016/S2213-8587(20)30129-7 PMC7437820

[53] Shafei A , Ramzy MM , Hegazy AI , et al. The molecular mechanisms of action of the endocrine disrupting chemical bisphenol A in the development of cancer. Gene. 2018;647:235‐243. 29317319 10.1016/j.gene.2018.01.016

[54] Leung YK , Govindarajah V , Cheong A , et al. Gestational high‐fat diet and bisphenol A exposure heightens mammary cancer risk. Endocr Relat Cancer. 2017;24(7):365‐378. 28487351 10.1530/ERC-17-0006 PMC5488396

[55] Darbre PD . Endocrine disruptors and obesity. Curr Obes Rep. 2017;6(1):18‐27. 28205155 10.1007/s13679-017-0240-4 PMC5359373

[56] Stern JH , Rutkowski JM , Scherer PE . Adiponectin, leptin, and fatty acids in the maintenance of metabolic homeostasis through adipose tissue crosstalk. Cell Metab. 2016;23(5):770‐784. 27166942 10.1016/j.cmet.2016.04.011 PMC4864949

[57] Sinha MK , Opentanova I , Ohannesian JP , et al. Evidence of free and bound leptin in human circulation. Studies in lean and obese subjects and during short‐term fasting. J Clin Invest. 1996;98(6):1277‐1282. 8823291 10.1172/JCI118913 PMC507552

[58] Saxena NK , Vertino PM , Anania FA , Sharma D . leptin‐induced growth stimulation of breast cancer cells involves recruitment of histone acetyltransferases and mediator complex to CYCLIN D1 promoter via activation of Stat3. J Biol Chem. 2007;282(18):13316‐13325. 17344214 10.1074/jbc.M609798200 PMC2923657

[59] Milosevic VS , Vukmirovic FC , Krstic MS , Zindovic MM , Lj Stojanovic D , Jancic SA . Involvement of leptin receptors expression in proliferation and neoangiogenesis in colorectal carcinoma. J buon. 2015;20(1):100‐108. 25778303

[60] Wei X , Liu Y , Gong C , et al. Targeting leptin as a therapeutic strategy against ovarian cancer peritoneal metastasis. Anticancer Agents Med Chem. 2017;17(8):1093‐1101. 28002999 10.2174/1871520616666161221114454

[61] Ray A , Cleary MP . The potential role of leptin in tumor invasion and metastasis. Cytokine Growth Factor Rev. 2017;38:80‐97. 29158066 10.1016/j.cytogfr.2017.11.002 PMC5720178

[62] Jiménez‐Cortegana C , López‐Saavedra A , Sánchez‐Jiménez F , et al. Leptin, both bad and good actor in cancer. Biomolecules. 2021;11(6):913. 10.3390/biom11060913 PMC8235379 34202969

[63] de Candia P , Prattichizzo F , Garavelli S , Alviggi C , La Cava A , Matarese G . The pleiotropic roles of leptin in metabolism, immunity, and cancer. J Exp Med. 2021;218(5):e20191593. 10.1084/jem.20191593 PMC8056770 33857282

[64] Mendoza‐Pérez J , Gu J , Herrera LA , et al. Prognostic significance of promoter CpG island methylation of obesity‐related genes in patients with nonmetastatic renal cell carcinoma. Cancer. 2017;123(18):3617‐3627. 28543182 10.1002/cncr.30707 PMC5589484

[65] Assidi M , Yahya FM , Al‐Zahrani MH , et al. Leptin protein expression and promoter methylation in ovarian cancer: a strong prognostic value with theranostic promises. Int J Mol Sci. 2021;22(23):12872. 10.3390/ijms222312872 PMC8657586 34884678

[66] Zhang TJ , Xu ZJ , Gu Y , et al. Identification and validation of obesity‐related gene LEP methylation as a prognostic indicator in patients with acute myeloid leukemia. Clin Epigenet. 2021;13(1):16. 10.1186/s13148-021-01013-9 PMC7824952 33485366

[67] Pasha HF , Mohamed RH , Toam MM , Yehia AM . Genetic and epigenetic modifications of adiponectin gene: potential association with breast cancer risk. J Gene Med. 2019;21(10):e3120. 31415715 10.1002/jgm.3120

[68] Parida S , Siddharth S , Sharma D . Adiponectin, obesity, and cancer: clash of the bigwigs in health and disease. Int J Mol Sci. 2019;20(10):2519. 10.3390/ijms20102519 PMC6566909 31121868

[69] Messaggio F . Increasing adiponectin receptor levels improves anti‐proliferative effects of adiporon in pancreatic cancer. Pancreas. 2017;46(10):1419.

[70] Cicekdal MB , Kazan BT , Tuna BG , et al. Effects of two types of energy restriction on methylation levels of adiponectin receptor 1 and leptin receptor overlapping transcript in a mouse mammary tumour virus‐transforming growth factor‐alpha breast cancer mouse model. Br J Nutr. 2021;125(1):1‐9. 31685042 10.1017/S0007114519002757

[71] Yamaji T , Iwasaki M , Sasazuki S , Tsugane S . Interaction between adiponectin and leptin influences the risk of colorectal adenoma. Cancer Res. 2010;70(13):5430‐5437. 20516125 10.1158/0008-5472.CAN-10-0178

[72] Baca P , Barajas‐Olmos F , Mirzaeicheshmeh E , et al. DNA methylation and gene expression analysis in adipose tissue to identify new loci associated with T2D development in obesity. Nutr Diabetes. 2022;12(1):50. 36535927 10.1038/s41387-022-00228-w PMC9763387

[73] Ouni M , Eichelmann F , Jähnert M , et al. Differences in DNA methylation of HAMP in blood cells predicts the development of type 2 diabetes. Mol Metab. 2023;75:101774. 37429525 10.1016/j.molmet.2023.101774 PMC10422014

[74] Touré M , Hichami A , Sayed A , Suliman M , Samb A , Khan NA . Association between polymorphisms and hypermethylation of CD36 gene in obese and obese diabetic Senegalese females. Diabetol Metab Syndr. 2022;14(1):117. 35982478 10.1186/s13098-022-00881-2 PMC9386198

[75] Krause C , Geißler C , Tackenberg H , et al. Multi‐layered epigenetic regulation of IRS2 expression in the liver of obese individuals with type 2 diabetes. Diabetologia. 2020;63(10):2182‐2193. 32710190 10.1007/s00125-020-05212-6 PMC7476982

[76] Wu L , Jiao Y , Li Y , et al. Hepatic Gadd45β promotes hyperglycemia and glucose intolerance through DNA demethylation of PGC‐1α. J Exp Med. 2021;218(5):e20201475. 10.1084/jem.20201475 PMC7953268 33688917

[77] Mirzaeicheshmeh E , Zerrweck C , Centeno‐Cruz F , et al. Alterations of DNA methylation during adipogenesis differentiation of mesenchymal stem cells isolated from adipose tissue of patients with obesity is associated with type 2 diabetes. Adipocyte. 2021;10(1):493‐504. 34699309 10.1080/21623945.2021.1978157 PMC8555535

[78] Schrader S , Perfilyev A , Ahlqvist E , et al. Novel subgroups of type 2 diabetes display different epigenetic patterns that associate with future diabetic complications. Diabetes Care. 2022;45(7):1621‐1630. 35607770 10.2337/dc21-2489 PMC9274219

[79] Veloso Pereira BM , Charleaux de Ponte M , Malavolta Luz AP , Thieme K . DNA methylation enzymes in the kidneys of male and female BTBR ob/ob mice. Front Endocrinol (Lausanne). 2023;14:1167546. 37091852 10.3389/fendo.2023.1167546 PMC10113614

[80] Yang L , Zhang Q , Wu Q , et al. Effect of TET2 on the pathogenesis of diabetic nephropathy through activation of transforming growth factor β1 expression via DNA demethylation. Life Sci. 2018;207:127‐137. 29705354 10.1016/j.lfs.2018.04.044

[81] Li KY , Tam CHT , Liu H , et al. DNA methylation markers for kidney function and progression of diabetic kidney disease. Nat Commun. 2023;14(1):2543. 37188670 10.1038/s41467-023-37837-7 PMC10185566

[82] Milluzzo A , Maugeri A , Barchitta M , Sciacca L , Agodi A . Epigenetic mechanisms in type 2 diabetes retinopathy: a systematic review. Int J Mol Sci. 2021;22(19):10502. 10.3390/ijms221910502 PMC8509039 34638838

[83] Rautenberg EK , Hamzaoui Y , Coletta DK . Mini‐review: mitochondrial DNA methylation in type 2 diabetes and obesity. Front Endocrinol (Lausanne). 2022;13:968268. 36093112 10.3389/fendo.2022.968268 PMC9453027

[84] Cao K , Lv W , Wang X , et al. Hypermethylation of hepatic mitochondrial ND6 provokes systemic insulin resistance. Adv Sci (Weinh). 2021;8(11):2004507. 34141522 10.1002/advs.202004507 PMC8188198

[85] Giglio RV , Stoian AP , Patti AM , et al. Genetic and epigenetic biomarkers for diagnosis, prognosis and treatment of metabolic syndrome. Curr Pharm Des. 2021;27(35):3729‐3740. 33845722 10.2174/1381612827666210412145915

[86] Masi S , Ambrosini S , Mohammed SA , et al. Epigenetic remodeling in obesity‐related vascular disease. Antioxid Redox Signal. 2021;34(15):1165‐1199. 32808539 10.1089/ars.2020.8040

[87] Sumi MP , Mahajan B , Sattar RSA , et al. Elucidation of epigenetic landscape in coronary artery disease: a review on basic concept to personalized medicine. Epigenet Insights. 2021;14:2516865720988567. 33598635 10.1177/2516865720988567 PMC7863167

[88] Do WL , Gohar J , McCullough LE , Galaviz KI , Conneely KN , Narayan KMV . Examining the association between adiposity and DNA methylation: a systematic review and meta‐analysis. Obes Rev. 2021;22(10):e13319. 34278703 10.1111/obr.13319

[89] Fernández‐Sanlés A , Sayols‐Baixeras S , Subirana I , Degano IR , Elosua R . Association between DNA methylation and coronary heart disease or other atherosclerotic events: a systematic review. Atherosclerosis. 2017;263:325‐333. 28577936 10.1016/j.atherosclerosis.2017.05.022

[90] Guo Y , Fan Y , Zhang J , et al. Perhexiline activates KLF14 and reduces atherosclerosis by modulating ApoA‐I production. J Clin Invest. 2015;125(10):3819‐3830. 26368306 10.1172/JCI79048 PMC4607137

[91] Wu S , Hsu LA , Teng MS , Chou HH , Ko YL . Differential genetic and epigenetic effects of the KLF14 gene on body shape indices and metabolic traits. Int J Mol Sci. 2022;23(8):4165. 10.3390/ijms23084165 PMC9032945 35456983

[92] Yeung BH , Griffiths K , Berger L , et al. Leptin induces epigenetic regulation of transient receptor potential melastatin 7 in rat adrenal pheochromocytoma cells. Am J Respir Cell Mol Biol. 2021;65(2):214‐221. 33891828 10.1165/rcmb.2020-0374OC PMC8399575

[93] Fontanella RA , Scisciola L , Rizzo MR , et al. Adiponectin related vascular and cardiac benefits in obesity: is there a role for an epigenetically regulated mechanism? Front Cardiovasc Med. 2021;8:768026. 34869683 10.3389/fcvm.2021.768026 PMC8639875

[94] Indumathi B , Oruganti SS , Sreenu B , Kutala VK . Association of promoter methylation and expression of inflammatory genes IL‐6 and TNF‐α with the risk of coronary artery disease in diabetic and obese subjects among Asian Indians. Indian J Clin Biochem. 2022;37(1):29‐39. 35125691 10.1007/s12291-020-00932-3 PMC8799818

[95] Geißler C , Krause C , Neumann AM , et al. Dietary induction of obesity and insulin resistance is associated with changes in Fgf21 DNA methylation in liver of mice. J Nutr Biochem. 2022;100:108907. 34801693 10.1016/j.jnutbio.2021.108907

[96] Kouzarides T . Chromatin modifications and their function. Cell. 2007;128(4):693‐705. 17320507 10.1016/j.cell.2007.02.005

[97] Zhang Y , Reinberg D . Transcription regulation by histone methylation: interplay between different covalent modifications of the core histone tails. Genes Dev. 2001;15(18):2343‐2360. 11562345 10.1101/gad.927301

[98] Labbe DP , Zadra G , Yang M , et al. High‐fat diet fuels prostate cancer progression by rewiring the metabolome and amplifying the MYC program. Nat Commun. 2019;10:4358. 31554818 10.1038/s41467-019-12298-z PMC6761092

[99] Wagner EJ , Carpenter PB . Understanding the language of Lys36 methylation at histone H3. Nat Rev Mol Cell Biol. 2012;13(2):115‐126. 22266761 10.1038/nrm3274 PMC3969746

[100] Li XJ , Li QL , Ju LG , et al. Deficiency of histone methyltransferase SET domain‐containing 2 in liver leads to abnormal lipid metabolism and HCC. Hepatology. 2021;73(5):1797‐1815. 33058300 10.1002/hep.31594

[101] Assante G , Chandrasekaran S , Ng S , et al. Acetyl‐CoA metabolism drives epigenome change and contributes to carcinogenesis risk in fatty liver disease. Genome Med. 2022;14(1):67. 10.1186/s13073-022-01071-5 PMC9219160 35739588

[102] Rajan PK , Utibe‐Abasi U , Sanabria JD , et al. The role of histone acetylation/methylation mediated epigenetic modifications in the pathogenesis of non‐alcoholic steatohepatitisassociated liver carcinogenesis. Am J Transplant. 2021;21(4):769.

[103] de Conti A , Dreval K , Tryndyak V , et al. Inhibition of the cell death pathway in nonalcoholic steatohepatitis (NASH)‐related hepatocarcinogenesis is associated with histone H4 lysine 16 deacetylation. Mol Cancer Res. 2017;15(9):1163‐1172. 28512251 10.1158/1541-7786.MCR-17-0109

[104] Qin Y , Roberts JD , Grimm SA , et al. An obesity‐associated gut microbiome reprograms the intestinal epigenome and leads to altered colonic gene expression. Genome Biol. 2018;19(1):7. 10.1186/s13059-018-1389-1 PMC5782396 29361968

[105] Porcuna J , Mínguez‐Martínez J , Ricote M . The PPARα and PPARγ epigenetic landscape in cancer and immune and metabolic disorders. Int J Mol Sci. 2021;22(19):10573. 10.3390/ijms221910573 PMC8508752 34638914

[106] Mayoral R , Osborn O , McNelis J , et al. Adipocyte SIRT1 knockout promotes PPARγ activity, adipogenesis and insulin sensitivity in chronic‐HFD and obesity. Mol Metab. 2015;4(5):378‐391. 25973386 10.1016/j.molmet.2015.02.007 PMC4421024

[107] Jiang X , Ye X , Guo W , Lu H , Gao Z . Inhibition of HDAC3 promotes ligand‐independent PPARγ activation by protein acetylation. J Mol Endocrinol. 2014;53(2):191‐200. 24982244 10.1530/JME-14-0066 PMC4391273

[108] Xu Z , Tong Q , Zhang Z , et al. Inhibition of HDAC3 prevents diabetic cardiomyopathy in OVE26 mice via epigenetic regulation of DUSP5‐ERK1/2 pathway. Clin Sci (Lond). 2017;131(15):1841‐1857. 28533215 10.1042/CS20170064 PMC5737625

[109] Zhang J , Xu Z , Gu J , et al. HDAC3 inhibition in diabetic mice may activate Nrf2 preventing diabetes‐induced liver damage and FGF21 synthesis and secretion leading to aortic protection. Am J Physiol Endocrinol Metab. 2018;315(2):E150‐E162. 29634312 10.1152/ajpendo.00465.2017

[110] Adhikari N , Jha T , Ghosh B . Dissecting histone deacetylase 3 in multiple disease conditions: selective inhibition as a promising therapeutic strategy. J Med Chem. 2021;64(13):8827‐8869. 34161101 10.1021/acs.jmedchem.0c01676

[111] Longo M , Zatterale F , Spinelli R , et al. Altered H3K4me3 profile at the TFAM promoter causes mitochondrial alterations in preadipocytes from first‐degree relatives of type 2 diabetics. Clin Epigenetics. 2023;15(1):144. 37679776 10.1186/s13148-023-01556-z PMC10486065

[112] Jetton TL , Flores‐Bringas P , Leahy JL , Gupta D . SetD7 (Set7/9) is a novel target of PPARγ that promotes the adaptive pancreatic β‐cell glycemic response. J Biol Chem. 2021;297(5):101250. 34592314 10.1016/j.jbc.2021.101250 PMC8526774

[113] Dubois‐Deruy E , El Masri Y , Turkieh A , Amouyel P , Pinet F , Annicotte JS . Cardiac acetylation in metabolic diseases. Biomedicines. 2022;10(8):1834. 10.3390/biomedicines10081834 PMC9405459 36009379

[114] Dhagia V , Kitagawa A , Jacob C , et al. G6PD activity contributes to the regulation of histone acetylation and gene expression in smooth muscle cells and to the pathogenesis of vascular diseases. Am J Physiol Heart Circ Physiol. 2021;320(3):H999‐H1016. 33416454 10.1152/ajpheart.00488.2020 PMC7988761

[115] Grootaert MOJ , Finigan A , Figg NL , Uryga AK , Bennett MR . SIRT6 protects smooth muscle cells from senescence and reduces atherosclerosis. Circ Res. 2021;128(4):474‐491. 33353368 10.1161/CIRCRESAHA.120.318353 PMC7899748

[116] Lecce L , Xu Y , V'Gangula B , et al. Histone deacetylase 9 promotes endothelial‐mesenchymal transition and an unfavorable atherosclerotic plaque phenotype. J Clin Invest. 2021;131(15):e131178. 10.1172/JCI131178 PMC8321575 34338228

[117] Ponting CP , Oliver PL , Reik W . Evolution and functions of long noncoding RNAs. Cell. 2009;136(4):629‐641. 19239885 10.1016/j.cell.2009.02.006

[118] Rey F , Messa L , Pandini C , et al. Transcriptome analysis of subcutaneous adipose tissue from severely obese patients highlights deregulation profiles in coding and non‐coding oncogenes. Int J Mol Sci. 2021;22(4):1989. 10.3390/ijms22041989 PMC7922682 33671464

[119] Tait S , Baldassarre A , Masotti A , et al. Integrated transcriptome analysis of human visceral adipocytes unravels dysregulated microRNA‐long non‐coding RNA‐mRNA networks in obesity and colorectal cancer. Front Oncol. 2020;10:1089. 32714872 10.3389/fonc.2020.01089 PMC7351520

[120] Cao XH , Yang K , Liang MX , et al. Variation of long non‐coding RNA and mRNA profiles in breast cancer cells with influences of adipocytes. Front Oncol. 2021;11:631551. 34094912 10.3389/fonc.2021.631551 PMC8176020

[121] De la Fuente‐Hernandez MA , Alanis‐Manriquez EC , Ferat‐Osorio E , et al. Molecular changes in adipocyte‐derived stem cells during their interplay with cervical cancer cells. Cell Oncol (Dordr). 2022;45(1):85‐101. 35013999 10.1007/s13402-021-00653-6 PMC12978079

[122] Wang LL , Wang RR , Ye Z , et al. PVT1 affects EMT and cell proliferation and migration via regulating p21 in triple‐negative breast cancer cells cultured with mature adipogenic medium. Acta Biochim Biophys Sin. 2018;50(12):1211‐1218. 30371726 10.1093/abbs/gmy129

[123] Choudhry H , Hassan MA , Al‐Malki AL , Al‐Sakkaf KA . Suppression of circulating AP001429.1 long non‐coding RNA in obese patients with breast cancer. Oncol Lett. 2021;22(1):508. 33986869 10.3892/ol.2021.12769 PMC8114468

[124] Wu H , Zhong Z , Wang A , et al. LncRNA FTX represses the progression of non‐alcoholic fatty liver disease to hepatocellular carcinoma via regulating the M1/M2 polarization of Kupffer cells. Cancer Cell Int. 2020;20:266. 32595415 10.1186/s12935-020-01354-0 PMC7315496

[125] Wang B , Li X , Hu W , Zhou Y , Din Y . Silencing of lncRNA SNHG20 delays the progression of nonalcoholic fatty liver disease to hepatocellular carcinoma via regulating liver Kupffer cells polarization. IUBMB Life. 2019;71(12):1952‐1961. 31408278 10.1002/iub.2137

[126] Shetty A , Suresh PS . A synergy of estradiol with leptin modulates the long non‐coding RNA NEAT1/mmu‐miR‐204‐5p/IGF1 axis in the uterus of high‐fat‐diet‐induced obese ovariectomized mice. J Steroid Biochem Mol Biol. 2021;209:105843. 33588025 10.1016/j.jsbmb.2021.105843

[127] Liu Y , Wang L , Liu H , Li C , He J . The prognostic significance of metabolic syndrome and a related six‐lncRNA signature in esophageal squamous cell carcinoma. Front Oncol. 2020;10:61. 32133283 10.3389/fonc.2020.00061 PMC7040247

[128] Liu Y , Li C , Fang L , et al. Lipid metabolism‐related lncRNA SLC25A21‐AS1 promotes the progression of oesophageal squamous cell carcinoma by regulating the NPM1/c‐Myc axis and SLC25A21 expression. Clin Transl Med. 2022;12(6):e944. 35735113 10.1002/ctm2.944 PMC9218933

[129] de Klerk JA , Beulens JWJ , Mei H , et al. Altered blood gene expression in the obesity‐related type 2 diabetes cluster may be causally involved in lipid metabolism: a Mendelian randomisation study. Diabetologia. 2023;66(6):1057‐1070. 36826505 10.1007/s00125-023-05886-8 PMC10163084

[130] Liu B , Zhong Y , Huang D , et al. LncRNA Nron deficiency protects mice from diet‐induced adiposity and hepatic steatosis. Metabolism. 2023;148:155609. 37277059 10.1016/j.metabol.2023.155609

[131] Kerr AG , Wang Z , Wang N , et al. The long noncoding RNA ADIPINT regulates human adipocyte metabolism via pyruvate carboxylase. Nat Commun. 2022;13(1):2958. 35618718 10.1038/s41467-022-30620-0 PMC9135762

[132] Alvarez‐Dominguez JR , Winther S , Hansen JB , Lodish HF , Knoll M . An adipose lncRAP2‐Igf2bp2 complex enhances adipogenesis and energy expenditure by stabilizing target mRNAs. iScience. 2022;25(1):103680. 35036870 10.1016/j.isci.2021.103680 PMC8749451

[133] Li Y , Chen Y , Liu Z , et al. Downregulation of Kcnq1ot1 attenuates β‐cell proliferation and insulin secretion via the miR‐15b‐5p/Ccnd1 and Ccnd2 axis. Acta Diabetol. 2022;59(7):885‐899. 35347427 10.1007/s00592-022-01871-6

[134] Zhang F , Yang Y , Chen X , et al. The long non‐coding RNA βFaar regulates islet β‐cell function and survival during obesity in mice. Nat Commun. 2021;12(1):3997. 34183666 10.1038/s41467-021-24302-6 PMC8238983

[135] Weiss E , Schrüfer A , Tocantins C , et al. Higher gestational weight gain delays wound healing and reduces expression of long non‐coding RNA KLRK1‐AS1 in neonatal endothelial progenitor cells. J Physiol. 2023;601(17):3961‐3974. 37470310 10.1113/JP284871 PMC10952284

[136] Zhao W , Yin Y , Cao H , Wang Y . Exercise improves endothelial function via the lncRNA MALAT1/miR‐320a axis in obese children and adolescents. Cardiol Res Pract. 2021;2021:8840698. 34123418 10.1155/2021/8840698 PMC8189819

[137] Li R , Yu X , Chen Y , et al. Association of lncRNA PVT1 gene polymorphisms with the risk of essential hypertension in chinese population. Biomed Res Int. 2022;2022:9976909. 35036445 10.1155/2022/9976909 PMC8758273

[138] Liu Y , Xu XY , Shen Y , et al. Ghrelin protects against obesity‐induced myocardial injury by regulating the lncRNA H19/miR‐29a/IGF‐1 signalling axis. Exp Mol Pathol. 2020;114:104405. 32084395 10.1016/j.yexmp.2020.104405

[139] Lang YY , Xu XY , Liu YL , et al. Ghrelin relieves obesity‐induced myocardial injury by regulating the epigenetic suppression of miR‐196b mediated by lncRNA HOTAIR. Obes Facts. 2022;15(4):540‐549. 35294947 10.1159/000523870 PMC9421679

[140] Heyn GS , Corrêa LH , Magalhães KG . The impact of adipose tissue‐derived miRNAs in metabolic syndrome, obesity, and cancer. Front Endocrinol (Lausanne). 2020;11:563816. 33123088 10.3389/fendo.2020.563816 PMC7573351

[141] Cheng L , Zhu Y , Han H , et al. MicroRNA‐148a deficiency promotes hepatic lipid metabolism and hepatocarcinogenesis in mice. Cell Death Dis. 2017;8(7):e2916. 28703810 10.1038/cddis.2017.309 PMC5550856

[142] Gjorgjieva M , Sobolewski C , Ay AS , et al. Genetic ablation of MiR‐22 fosters diet‐induced obesity and NAFLD development. J Pers Med. 2020;10(4):170. 10.3390/jpm10040170 PMC7711493 33066497

[143] Feige JN , Gelman L , Michalik L , Desvergne B , Wahli W . From molecular action to physiological outputs: peroxisome proliferator‐activated receptors are nuclear receptors at the crossroads of key cellular functions. Prog Lipid Res. 2006;45(2):120‐159. 16476485 10.1016/j.plipres.2005.12.002

[144] Hsu HT , Sung MT , Lee CC , et al. Peroxisome proliferator‐activated receptor γ expression is inversely associated with macroscopic vascular invasion in human hepatocellular carcinoma. Int J Mol Sci. 2016;17(8):1226. 10.3390/ijms17081226 PMC5000624 27483249

[145] Motawi TK , Shaker OG , Ismail MF , Sayed NH . Peroxisome proliferator‐activated receptor gamma in obesity and colorectal cancer: the role of epigenetics. Sci Rep. 2017;7(1):10714. 28878369 10.1038/s41598-017-11180-6 PMC5587696

[146] Rajarajan D , Selvarajan S , Charan Raja MR , Kar Mahapatra S , Kasiappan R . Genome‐wide analysis reveals miR‐3184‐5p and miR‐181c‐3p as a critical regulator for adipocytes‐associated breast cancer. J Cell Physiol. 2019;234(10):17959‐17974. 30847933 10.1002/jcp.28428

[147] Belaiba F , Medimegh I , Ammar M , et al. Expression and polymorphism of micro‐RNA according to body mass index and breast cancer presentation in Tunisian patients. J Leukoc Biol. 2019;105(2):317‐327. 30303554 10.1002/JLB.3VMA0618-218R

[148] Meerson A , Eliraz Y , Yehuda H , et al. Obesity impacts the regulation of miR‐10b and its targets in primary breast tumors. BMC Cancer. 2019;19(1):86. 30658617 10.1186/s12885-019-5300-6 PMC6339293

[149] Ahmed FE . miRNA as markers for the diagnostic screening of colon cancer. Expert Rev Anticancer Ther. 2014;14(4):463‐485. 24580550 10.1586/14737140.2014.869479

[150] Jeon J , Olkhov‐Mitsel E , Xie H , et al. Temporal stability and prognostic biomarker potential of the prostate cancer urine miRNA transcriptome. J Natl Cancer Inst. 2020;112(3):247‐255. 31161221 10.1093/jnci/djz112 PMC7073919

[151] Donkers H , Hirschfeld M , Weiss D , et al. Detection of microRNA in urine to identify patients with endometrial cancer: a feasibility study. Int J Gynecol Cancer. 2021;31(6):868‐874. 33911004 10.1136/ijgc-2021-002494

[152] Jasinski‐Bergner S , Kielstein H . Adipokines regulate the expression of tumor‐relevant microRNAs. Obesity Facts. 2019;12(2):211‐225. 30999294 10.1159/000496625 PMC6547259

[153] Nagalingam A , Siddharth S , Parida S , et al. Hyperleptinemia in obese state renders luminal breast cancers refractory to tamoxifen by coordinating a crosstalk between Med1, miR205 and ErbB. Npj Breast Cancer. 2021;7(1):105. 10.1038/s41523-021-00314-9 PMC8363746 34389732

[154] Rios‐Colon L , Chijioke J , Niture S , et al. Leptin modulated microRNA‐628‐5p targets Jagged‐1 and inhibits prostate cancer hallmarks. Sci Rep. 2022;12(1). 10.1038/s41598-022-13279-x PMC9203512 35710817

[155] Ma L , Fan Z , Du G , Wang H . Leptin‐elicited miRNA‐342‐3p potentiates gemcitabine resistance in pancreatic ductal adenocarcinoma. Biochem Biophys Res Commun. 2019;509(3):845‐853. 30638935 10.1016/j.bbrc.2019.01.030

[156] Al‐Khalaf HH , Amir M , Al‐Mohanna F , Tulbah A , Al‐Sayed A , Aboussekhra A . Obesity and p16(INK4A) downregulation activate breast adipocytes and promote their protumorigenicity. Mol Cell Biol. 2017;37(17):e00101‐17. 10.1128/MCB.00101-17 PMC5559683 28630279

[157] Moraes JA , Encarnação C , Franco VA , et al. Adipose tissue‐derived extracellular vesicles and the tumor microenvironment: revisiting the hallmarks of cancer. Cancers (Basel). 2021;13(13):3328. 10.3390/cancers13133328 PMC8268128 34283044

[158] Liu Y , Tan J , Ou S , Chen J , Chen L . Adipose‐derived exosomes deliver miR‐23a/b to regulate tumor growth in hepatocellular cancer by targeting the VHL/HIF axis. J Physiol Biochem. 2019;75(3):391‐401. 31321740 10.1007/s13105-019-00692-6

[159] Wu Q , Sun S , Li Z , et al. Breast cancer‐released exosomes trigger cancer‐associated cachexia to promote tumor progression. Adipocyte. 2019;8(1):31‐45. 30474469 10.1080/21623945.2018.1551688 PMC6768245

[160] Cao M , Isaac R , Yan W , et al. Cancer‐cell‐secreted extracellular vesicles suppress insulin secretion through miR‐122 to impair systemic glucose homeostasis and contribute to tumour growth. Nat Cell Biol. 2022;24(6):954‐967. 35637408 10.1038/s41556-022-00919-7 PMC9233030

[161] Cariello M , Piccinin E , Pasculli E , et al. Platelets from patients with visceral obesity promote colon cancer growth. Commun Biol. 2022;5(1). 10.1038/s42003-022-03486-7 PMC9174292 35672444

[162] Cirillo F , Catellani C , Sartori C , Lazzeroni P , Amarri S , Street ME . Obesity, insulin resistance, and colorectal cancer: could miRNA dysregulation play a role? Int J Mol Sci. 2019;20(12):2922. 10.3390/ijms20122922 PMC6628223 31207998

[163] Taroeno‐Hariadi KW , Hardianti MS , Sinorita H , Aryandono T . Obesity, leptin, and deregulation of microRNA in lipid metabolisms: their contribution to breast cancer prognosis. Diabetol Metab Syndr. 2021;13(1):10. 33482868 10.1186/s13098-020-00621-4 PMC7821690

[164] Lou G , Song X , Yang F , et al. Exosomes derived from miR‐122‐modified adipose tissue‐derived MSCs increase chemosensitivity of hepatocellular carcinoma. J Hematol Oncol. 2015;8:122. 26514126 10.1186/s13045-015-0220-7 PMC4627430

[165] Sheykhhasan M , Kalhor N , Sheikholeslami A , Dolati M , Amini E , Fazaeli H . Exosomes of mesenchymal stem cells as a proper vehicle for transfecting miR‐145 into the breast cancer cell line and its effect on metastasis. Biomed Res Int. 2021;2021:5516078. 34307654 10.1155/2021/5516078 PMC8263260

[166] Lou G , Chen L , Xia C , et al. MiR‐199a‐modified exosomes from adipose tissue‐derived mesenchymal stem cells improve hepatocellular carcinoma chemosensitivity through mTOR pathway. J Exp Clin Cancer Res. 2020;39(1):4. 31898515 10.1186/s13046-019-1512-5 PMC6941283

[167] Zhou Y , Yamamoto Y , Takeshita F , Yamamoto T , Xiao Z , Ochiya T . Delivery of miR‐424‐5p via extracellular vesicles promotes the apoptosis of MDA‐MB‐231 TNBC cells in the tumor microenvironment. Int J Mol Sci. 2021;22(2):844. 10.3390/ijms22020844 PMC7831022 33467725

[168] Santos D , Porter‐Gill P , Goode G , et al. Circulating microRNA levels differ in the early stages of insulin resistance in prepubertal children with obesity. Life Sci. 2023;312:121246. 36455651 10.1016/j.lfs.2022.121246 PMC10375861

[169] Zhu J , Wang C , Zhang X , et al. Correlation analysis of microribonucleic acid‐155 and microribonucleic acid‐29 with type 2 diabetes mellitus, and the prediction and verification of target genes. J Diabetes Investig. 2021;12(2):165‐175. 10.1111/jdi.13334 PMC7858142 32579760

[170] Chu X , Hou Y , Zhang X , et al. Hepatic glucose metabolism disorder induced by adipose tissue‐derived miR‐548ag via DPP4 upregulation. Int J Mol Sci. 2023;24(3):2964. 10.3390/ijms24032964 PMC9917501 36769291

[171] Catanzaro G , Conte F , Trocchianesi S , et al. Network analysis identifies circulating miR‐155 as predictive biomarker of type 2 diabetes mellitus development in obese patients: a pilot study. Sci Rep. 2023;13(1):19496. 37945677 10.1038/s41598-023-46516-y PMC10636008

[172] Dracheva KV , Pobozheva IA , Anisimova KA , et al. Downregulation of exosomal hsa‐miR‐551b‐3p in obesity and its link to type 2 diabetes mellitus. Noncoding RNA. 2023;9(6):67. 10.3390/ncrna9060067 PMC10660712 37987363

[173] Li JM , Li X , Chan LWC , et al. Lipotoxicity‐polarised macrophage‐derived exosomes regulate mitochondrial fitness through Miro1‐mediated mitophagy inhibition and contribute to type 2 diabetes development in mice. Diabetologia. 2023;66(12):2368‐2386. 37615690 10.1007/s00125-023-05992-7

[174] Shan Z , Fa WH , Tian CR , Yuan CS , Jie N . Mitophagy and mitochondrial dynamics in type 2 diabetes mellitus treatment. Aging (Albany NY). 2022;14(6):2902‐2919. 35332108 10.18632/aging.203969 PMC9004550

[175] Karere GM , Glenn JP , Li G , Konar A , VandeBerg JL , Cox LA . Potential miRNA biomarkers and therapeutic targets for early atherosclerotic lesions. Sci Rep. 2023;13(1):3467. 36859458 10.1038/s41598-023-29074-1 PMC9977938

[176] Elmoselhi AB , Seif Allah M , Bouzid A , et al. Circulating microRNAs as potential biomarkers of early vascular damage in vitamin D deficiency, obese, and diabetic patients. PLoS One. 2023;18(3):e0283608. 36952563 10.1371/journal.pone.0283608 PMC10035929

[177] Wan F , Ma X , Wang J , An Z , Xue J , Wang Q . Evaluation of left ventricular dysfunction by three‐dimensional speckle‐tracking echocardiography and bioinformatics analysis of circulating exosomal miRNA in obese patients. BMC Cardiovasc Disord. 2023;23(1):450. 37697228 10.1186/s12872-023-03502-6 PMC10496196

[178] Dogan N , Ozuynuk‐Ertugrul AS , Balkanay OO , et al. Examining the effects of coronary artery disease‐ and mitochondrial biogenesis‐related genes' and microRNAs' expression levels on metabolic disorders in epicardial adipose tissue. Gene. 2023;895:147988. 37977322 10.1016/j.gene.2023.147988

[179] Tang Y , Yang LJ , Liu H , et al. Exosomal miR‐27b‐3p secreted by visceral adipocytes contributes to endothelial inflammation and atherogenesis. Cell Rep. 2023;42(1):111948. 36640325 10.1016/j.celrep.2022.111948

[180] Salmena L , Poliseno L , Tay Y , Kats L , Pandolfi PP . A ceRNA hypothesis: the Rosetta Stone of a hidden RNA language? Cell. 2011;146(3):353‐358. 21802130 10.1016/j.cell.2011.07.014 PMC3235919

[181] Kristensen LS , Andersen MS , Stagsted LVW , Ebbesen KK , Hansen TB , Kjems J . The biogenesis, biology and characterization of circular RNAs. Nat Rev Genet. 2019;20(11):675‐691. 31395983 10.1038/s41576-019-0158-7

[182] Zhang Y , Tian Z , Ye H , et al. Emerging functions of circular RNA in the regulation of adipocyte metabolism and obesity. Cell Death Discov. 2022;8(1):268. 35595755 10.1038/s41420-022-01062-w PMC9122900

[183] Chen C , Zhang X , Deng Y , et al. Regulatory roles of circRNAs in adipogenesis and lipid metabolism: emerging insights into lipid‐related diseases. FEBS J. 2021;288(12):3663‐3682. 32798313 10.1111/febs.15525

[184] Li G , Zhang H , Cao K , et al. Transcriptome of visceral adipose tissue identifies an inflammation‐related ceRNA network that regulates obesity. Mol Cell Biochem. 2022;;477(4):1095‐1106. 35064875 10.1007/s11010-022-04362-y

[185] Zhang Z , Zhang T , Feng R , Huang H , Xia T , Sun C . circARF3 alleviates mitophagy‐mediated inflammation by targeting miR‐103/TRAF3 in mouse adipose tissue. Mol Ther Nucleic Acids. 2019;14:192‐203. 30623853 10.1016/j.omtn.2018.11.014 PMC6325073

[186] Zhang PP , Han Q , Sheng MX , et al. Identification of circular RNA expression profiles in white adipocytes and their roles in adipogenesis. Front Physiol. 2021;12:728208. 34489740 10.3389/fphys.2021.728208 PMC8417237

[187] Zhang P , Sheng M , Du C , et al. Assessment of CircRNA expression profiles and potential functions in brown adipogenesis. Front Genet. 2021;12:769690. 34745232 10.3389/fgene.2021.769690 PMC8569449

[188] Liu Y , Liu H , Li Y , et al. Circular RNA SAMD4A controls adipogenesis in obesity through the miR‐138‐5p/EZH2 axis. Theranostics. 2020;10(10):4705‐4719. 32292524 10.7150/thno.42417 PMC7150479

[189] Arcinas C , Tan W , Fang W , et al. Adipose circular RNAs exhibit dynamic regulation in obesity and functional role in adipogenesis. Nat Metab. 2019;1(7):688‐703. 32694641 10.1038/s42255-019-0078-z

[190] Firoozi Z , Mohammadisoleimani E , Shahi A , et al. Potential roles of hsa_circ_000839 and hsa_circ_0005986 in breast cancer. J Clin Lab Anal. 2022;36(3): e24263. 10.1002/jcla.24263 PMC8906031 35098570

[191] Takenaka K , Olzomer EM , Hoehn KL , et al. Investigation of circular RNA transcriptome in obesity‐related endometrial cancer. Gene. 2023;855:147125. 36549426 10.1016/j.gene.2022.147125

[192] Li Y , Jiang B , Zeng L , et al. Adipocyte‐derived exosomes promote the progression of triple‐negative breast cancer through circCRIM1‐dependent OGA activation. Environ Res. 2023:117266. 37775001 10.1016/j.envres.2023.117266

[193] Dai Y , Ma X , Zhang J , Yu S , Zhu Y , Wang J . hsa_circ_0115355 promotes pancreatic β‐cell function in patients with type 2 diabetes through the miR‐145/SIRT1 axis. J Clin Lab Anal. 2022;36(8):e24583. 35778952 10.1002/jcla.24583 PMC9396171

[194] Ma J , Wu Y , He Y . Silencing circRNA LRP6 down‐regulates PRMT1 to improve the streptozocin‐induced pancreatic β‐cell injury and insulin secretion by sponging miR‐9‐5p. J Bioenerg Biomembr. 2021;53(3):333‐342. 33826088 10.1007/s10863-021-09895-3

[195] Teaney NA , Cyr NE . FoxO1 as a tissue‐specific therapeutic target for type 2 diabetes. Front Endocrinol (Lausanne). 2023;14:1286838. 37941908 10.3389/fendo.2023.1286838 PMC10629996

[196] Cai H , Jiang Z , Yang X , Lin J , Cai Q , Li X . Circular RNA HIPK3 contributes to hyperglycemia and insulin homeostasis by sponging miR‐192‐5p and upregulating transcription factor forkhead box O1. Endocr J. 2020;67(4):397‐408. 31875589 10.1507/endocrj.EJ19-0271

[197] Dandare A , Khan MJ , Naeem A , Liaquat A . Clinical relevance of circulating non‐coding RNAs in metabolic diseases: emphasis on obesity, diabetes, cardiovascular diseases and metabolic syndrome. Genes Dis. 2023;10(6):2393‐2413. 37554181 10.1016/j.gendis.2022.05.022 PMC10404886

[198] Zhao Z , Li X , Gao C , et al. Peripheral blood circular RNA hsa_circ_0124644 can be used as a diagnostic biomarker of coronary artery disease. Sci Rep. 2017;7:39918. 28045102 10.1038/srep39918 PMC5206672

[199] Wen C , Li B , Nie L , Mao L , Xia Y . Emerging roles of extracellular vesicle‐delivered circular RNAs in atherosclerosis. Front Cell Dev Biol. 2022;10:804247. 35445015 10.3389/fcell.2022.804247 PMC9014218

[200] Clapier CR , Cairns BR . The biology of chromatin remodeling complexes. Annu Rev Biochem. 2009;78:273‐304. 19355820 10.1146/annurev.biochem.77.062706.153223

[201] Clapier CR , Iwasa J , Cairns BR , Peterson CL . Mechanisms of action and regulation of ATP‐dependent chromatin‐remodelling complexes. Nat Rev Mol Cell Biol. 2017;18(7):407‐422. 28512350 10.1038/nrm.2017.26 PMC8127953

[202] Ebot EM , Gerke T , Labbé DP , et al. Gene expression profiling of prostate tissue identifies chromatin regulation as a potential link between obesity and lethal prostate cancer. Cancer. 2017;123(21):4130‐4138. 28700821 10.1002/cncr.30831 PMC5802874

[203] Pant R , Alam A , Choksi A , Shah VK , Firmal P , Chattopadhyay S . Chromatin remodeling protein SMAR1 regulates adipogenesis by modulating the expression of PPAR gamma. Biochim Biophys Acta Mol Cell Biol Lipids. 2021;1866(12):159045. 10.1016/j.bbalip.2021.159045 34450266

[204] Taye N , Alam A , Ghorai S , et al. SMAR1 inhibits Wnt/β‐catenin signaling and prevents colorectal cancer progression. Oncotarget. 2018;9(30):21322‐21336. 29765542 10.18632/oncotarget.25093 PMC5940383

[205] Moore A , Wu L , Chuang J‐C , et al. Arid1a loss drives nonalcoholic steatohepatitis in mice through epigenetic dysregulation of hepatic lipogenesis and fatty acid oxidation. Hepatology. 2019;69(5):1931‐1945. 30584660 10.1002/hep.30487 PMC6461494

[206] Sun X , Wang SC , Wei Y , et al. Arid1a has context‐dependent oncogenic and tumor suppressor functions in liver cancer. Cancer Cell. 2017;32(5):574‐589. e6. 29136504 10.1016/j.ccell.2017.10.007 PMC5728182

[207] Yang P‐B , Hou P‐P , Liu F‐Y , et al. Blocking PPAR gamma interaction facilitates Nur77 interdiction of fatty acid uptake and suppresses breast cancer progression. Proc Natl Acad Sci USA. 2020;117(44):27412‐27422. 33087562 10.1073/pnas.2002997117 PMC7959534

[208] Liu XZ , Rulina A , Choi MH , et al. C/EBPB‐dependent adaptation to palmitic acid promotes tumor formation in hormone receptor negative breast cancer. Nat Commun. 2022;13(1):69. 35013251 10.1038/s41467-021-27734-2 PMC8748947

[209] Wei Z , Yoshihara E , He N , et al. Vitamin D switches BAF complexes to protect β cells. Cell. 2018;173(5):1135‐1149. e15. 29754817 10.1016/j.cell.2018.04.013 PMC5987229

[210] Wang RR , Qiu X , Pan R , et al. Dietary intervention preserves β cell function in mice through CTCF‐mediated transcriptional reprogramming. J Exp Med. 2022;219(7):e20211779. 10.1084/jem.20211779 PMC9166293 35652891

[211] Kong Q , Zou J , Zhang Z , et al. BAF60a deficiency in macrophage promotes diet‐induced obesity and metabolic inflammation. Diabetes. 2022;71(10):2136‐2152. 35822944 10.2337/db22-0114

[212] Iyengar NM , Gucalp A , Dannenberg AJ , Hudis CA . Obesity and cancer mechanisms: tumor microenvironment and inflammation. J Clin Oncol. 2016;34(35):4270‐4276. 27903155 10.1200/JCO.2016.67.4283 PMC5562428

[213] Cani PD , Jordan BF . Gut microbiota‐mediated inflammation in obesity: a link with gastrointestinal cancer. Nat Rev Gastroenterol Hepatol. 2018;15(11):671‐682. 29844585 10.1038/s41575-018-0025-6

[214] Weisberg SP , McCann D , Desai M , Rosenbaum M , Leibel RL . Obesity is associated with macrophage accumulation in adipose tissue. J Clin Invest. 2003;112(12):1796‐1808. 14679176 10.1172/JCI19246 PMC296995

[215] Harman‐Boehm I , Blüher M , Redel H , et al. Macrophage infiltration into omental versus subcutaneous fat across different populations: effect of regional adiposity and the comorbidities of obesity. J Clin Endocrinol Metab. 2007;92(6):2240‐2247. 17374712 10.1210/jc.2006-1811

[216] Iyengar NM , Zhou XK , Gucalp A , et al. Systemic correlates of white adipose tissue inflammation in early‐stage breast cancer. Clin Cancer Res. 2016;22(9):2283‐2289. 26712688 10.1158/1078-0432.CCR-15-2239 PMC4854755

[217] Gucalp A , Iyengar NM , Zhou XK , et al. Periprostatic adipose inflammation is associated with high‐grade prostate cancer. Prostate Cancer Prostatic Dis. 2017;20(4):418‐423. 28653675 10.1038/pcan.2017.31 PMC5681425

[218] Ley K . M1 means kill; M2 means heal. J Immunol. 2017;199(7):2191‐2193. 28923980 10.4049/jimmunol.1701135

[219] Lumeng CN , Bodzin JL , Saltiel AR . Obesity induces a phenotypic switch in adipose tissue macrophage polarization. J Clin Invest. 2007;117(1):175‐184. 17200717 10.1172/JCI29881 PMC1716210

[220] Kern L , Mittenbühler MJ , Vesting AJ , Ostermann AL , Wunderlich CM , Wunderlich FT . Obesity‐induced TNFα and IL‐6 signaling: the missing link between obesity and inflammation‐driven liver and colorectal cancers. Cancers (Basel). 2018;11(1):24. 10.3390/cancers11010024 PMC6356226 30591653

[221] Liu X‐L , Pan Q , Cao H‐X , et al. Lipotoxic hepatocyte‐derived exosomal MicroRNA 192–5p activates macrophages through rictor/Akt/Forkhead box transcription factor O1 signaling in nonalcoholic fatty liver disease. Hepatology. 2020;72(2):454‐469. 31782176 10.1002/hep.31050 PMC10465073

[222] Liu H , Niu Q , Wang T , Dong H , Bian C . Lipotoxic hepatocytes promote nonalcoholic fatty liver disease progression by delivering microRNA‐9‐5p and activating macrophages. Int J Biol Sci. 2021;17(14):3745‐3759. 35261562 10.7150/ijbs.57610 PMC8898344

[223] Phu TA , Ng M , Vu NK , Bouchareychas L , Raffai RL . IL‐4 polarized human macrophage exosomes control cardiometabolic inflammation and diabetes in obesity. Mol Ther. 2022;30(6):2274‐2297. 35292359 10.1016/j.ymthe.2022.03.008 PMC9171286

[224] Li L , Zuo H , Huang X , et al. Bone marrow macrophage‐derived exosomal miR‐143‐5p contributes to insulin resistance in hepatocytes by repressing MKP5. Cell Proliferation. 2021;54(12):e13140. 10.1111/cpr.13140 PMC8666281 34647385

[225] Li D , Yang C , Zhu J‐Z , et al. Berberine remodels adipose tissue to attenuate metabolic disorders by activating sirtuin 3. Acta Pharmacol Sin. 2021;43(5):1285‐1298. 10.1038/s41401-021-00736-y PMC9061715 34417576

[226] Han Y‐B , Tian M , Wang X‐X , et al. Berberine ameliorates obesity‐induced chronic inflammation through suppression of ER stress and promotion of macrophage M2 polarization at least partly via downregulating lncRNA Gomafu. Int Immunopharmacol. 2020;86:106741. 32650294 10.1016/j.intimp.2020.106741

[227] Chen L , Zhang J , Zou Y , et al. Kdm2a deficiency in macrophages enhances thermogenesis to protect mice against HFD‐induced obesity by enhancing H3K36me2 at the Pparg locus. Cell Death Different. 2021;28(6):1880‐1899. 10.1038/s41418-020-00714-7 PMC8185071 33462408

[228] Sprenkle NT , Winn NC , Bunn KE , et al. The miR‐23‐27‐24 clusters drive lipid‐associated macrophage proliferation in obese adipose tissue. Cell Rep. 2023;42(8):112928. 37542720 10.1016/j.celrep.2023.112928 PMC10712211

[229] Ying W , Gao H , Dos Reis FCG , et al. MiR‐690, an exosomal‐derived miRNA from M2‐polarized macrophages, improves insulin sensitivity in obese mice. Cell Metabolism. 2021;33(4):781. 33450179 10.1016/j.cmet.2020.12.019 PMC8035248

[230] Quail DF , Dannenberg AJ . The obese adipose tissue microenvironment in cancer development and progression. Nat Rev Endocrinol. 2019;15(3):139‐154. 30459447 10.1038/s41574-018-0126-x PMC6374176

[231] Wang Z , Aguilar EG , Luna JI , et al. Paradoxical effects of obesity on T cell function during tumor progression and PD‐1 checkpoint blockade. Nat Med. 2019;25(1):141‐151. 30420753 10.1038/s41591-018-0221-5 PMC6324991

[232] Zhou D , Hlady RA , Schafer MJ , et al. High fat diet and exercise lead to a disrupted and pathogenic DNA methylome in mouse liver. Epigenetics. 2017;12(1):55‐69. 27858497 10.1080/15592294.2016.1261239 PMC5270632

[233] Prasad M , Rajagopal P , Devarajan N , et al. A comprehensive review on high ‐fat diet‐induced diabetes mellitus: an epigenetic view. J Nutr Biochem. 2022;107:109037. 35533900 10.1016/j.jnutbio.2022.109037

[234] Viraragavan A , Willmer T , Patel O , Basson A , Johnson R , Pheiffer C . Cafeteria diet induces global and Slc27a3‐specific hypomethylation in male Wistar rats. Adipocyte. 2021;10(1):108‐118. 33570456 10.1080/21623945.2021.1886697 PMC7889207

[235] Romagnolo DF , Donovan MG , Doetschman TC , Selmin OI . N‐6 linoleic acid induces epigenetics alterations associated with colonic inflammation and cancer. Nutrients. 2019;11(1):171. 10.3390/nu11010171 PMC6356359 30650553

[236] Wahab NA , Elias MH , Ali RAR , Mokhtar NM . Chronic consumption of fructose dysregulates genes related to glucose and lipid metabolism in prostate tissue. Sains Malaysiana. 2018;47(10):2501‐2507.

[237] Petito G , Giacco A , Cioffi F , et al. Short‐term fructose feeding alters tissue metabolic pathways by modulating microRNAs expression both in young and adult rats. Front Cell Dev Biol. 2023;11:1101844. 36875756 10.3389/fcell.2023.1101844 PMC9977821

[238] Li X , Shao X , Bazzano LA , et al. Blood DNA methylation at TXNIP and glycemic changes in response to weight‐loss diet interventions: the POUNDS lost trial. Int J Obes (Lond). 2022;46(6):1122‐1127. 35165382 10.1038/s41366-022-01084-5 PMC9156542

[239] Domínguez‐Barragán J , Fernández‐Sanlés A , Hernáez Á , et al. Blood DNA methylation signature of diet quality, and association with cardiometabolic traits. Eur J Prev Cardiol. 2023;31(2):191‐202. 10.1093/eurjpc/zwad317 PMC10809172 37793095

[240] Joshi T , Patel I , Kumar A , Donovan V , Levenson AS . Grape powder supplementation attenuates prostate neoplasia associated with pten haploinsufficiency in mice fed high‐fat diet. Mol Nutr Food Res. 2020;64(16):e2000326. 32618118 10.1002/mnfr.202000326 PMC8103660

[241] Li Y , Chen M , Tollefsbol T . The epigenetic influence of maternal dieton prevention of high‐fat diet induced obesity and breastcancer in later life. Cancer Res. 2020;80(16):2440.

[242] Chen M , Li S , Arora I , et al. Maternal soybean diet on prevention of obesity‐related breast cancer through early‐life gut microbiome and epigenetic regulation. J Nutr Biochem. 2022;110:109119. 35933021 10.1016/j.jnutbio.2022.109119 PMC9792070

[243] Deng X , Liang J , Wang L , et al. Whole grain proso millet (Panicum miliaceum L.) attenuates hyperglycemia in type 2 diabetic mice: involvement of miRNA profile. J Agric Food Chem. 2023;71(24):9324‐9336. 37294881 10.1021/acs.jafc.2c08184

[244] Jiang Y , Sun‐Waterhouse D , Chen Y , Li F , Li D . Epigenetic mechanisms underlying the benefits of flavonoids in cardiovascular health and diseases: are long non‐coding RNAs rising stars? Crit Rev Food Sci Nutr. 2022;62(14):3855‐3872. 33427492 10.1080/10408398.2020.1870926

[245] Potenza MA , Iacobazzi D , Sgarra L , Montagnani M . The intrinsic virtues of EGCG, an extremely good cell guardian, on prevention and treatment of diabesity complications. Molecules. 2020;25(13):3061. 10.3390/molecules25133061 PMC7411588 32635492

[246] Mishra SP , Wang B , Jain S , et al. A mechanism by which gut microbiota elevates permeability and inflammation in obese/diabetic mice and human gut. Gut. 2023;72(10):1848‐1865. 36948576 10.1136/gutjnl-2022-327365 PMC10512000

[247] Okuka N , Schuh V , Krammer U , et al. Epigenetic aspects of a new probiotic concept—a pilot study. Life (Basel). 2023;13(9):1912. 10.3390/life13091912 PMC10533075 37763315

[248] Gershuni VM , Yan SL , Medici V . Nutritional ketosis for weight management and reversal of metabolic syndrome. Curr Nutr Rep. 2018;7(3):97‐106. 30128963 10.1007/s13668-018-0235-0 PMC6472268

[249] Choi YJ , Jeon SM , Shin S . Impact of a ketogenic diet on metabolic parameters in patients with obesity or overweight and with or without type 2 diabetes: a meta‐analysis of randomized controlled trials. Nutrients. 2020;12(7):2005. 10.3390/nu12072005 PMC7400909 32640608

[250] Weber DD , Aminzadeh‐Gohari S , Tulipan J , Catalano L , Feichtinger RG , Kofler B . Ketogenic diet in the treatment of cancer—Where do we stand? Mol Metab. 2020;33:102‐121. 31399389 10.1016/j.molmet.2019.06.026 PMC7056920

[251] Ellenbroek JH , van Dijck L , Töns HA , et al. Long‐term ketogenic diet causes glucose intolerance and reduced β‐ and α‐cell mass but no weight loss in mice. Am J Physiol Endocrinol Metab. 2014;306(5):E552‐E558. 24398402 10.1152/ajpendo.00453.2013

[252] O'Neill B , Raggi P . The ketogenic diet: pros and cons. Atherosclerosis. 2020;292:119‐126. 31805451 10.1016/j.atherosclerosis.2019.11.021

[253] Dmitrieva‐Posocco O , Wong AC , Lundgren P , et al. β‐Hydroxybutyrate suppresses colorectal cancer. Nature. 2022;605(7908):160‐165. 35477756 10.1038/s41586-022-04649-6 PMC9448510

[254] Xie Z , Zhang D , Chung D , et al. Metabolic regulation of gene expression by histone lysine β‐hydroxybutyrylation. Mol Cell. 2016;62(2):194‐206. 27105115 10.1016/j.molcel.2016.03.036 PMC5540445

[255] Zhang H , Chang Z , Qin LN , et al. MTA2 triggered R‐loop trans‐regulates BDH1‐mediated β‐hydroxybutyrylation and potentiates propagation of hepatocellular carcinoma stem cells. Signal Transduct Target Ther. 2021;6(1):135. 33795651 10.1038/s41392-021-00464-z PMC8016859

[256] Liu K , Li F , Sun Q , et al. p53 β‐hydroxybutyrylation attenuates p53 activity. Cell Death Dis. 2019;10(3):243. 30858356 10.1038/s41419-019-1463-y PMC6411878

[257] Koronowski KB , Greco CM , Huang H , et al. Ketogenesis impact on liver metabolism revealed by proteomics of lysine β‐hydroxybutyrylation. Cell Rep. 2021;36(5):109487. 34348140 10.1016/j.celrep.2021.109487 PMC8372761

[258] Ferrer M , Mourikis N , Davidson EE , et al. Ketogenic diet promotes tumor ferroptosis but induces relative corticosterone deficiency that accelerates cachexia. Cell Metab. 2023; doi: 10.1101/2023.02.17.528937. 10.1016/j.cmet.2023.05.008 PMC11037504 37311455

[259] Friedenreich CM , Ryder‐Burbidge C , McNeil J . Physical activity, obesity and sedentary behavior in cancer etiology: epidemiologic evidence and biologic mechanisms. Mol Oncol. 2021;15(3):790‐800. 32741068 10.1002/1878-0261.12772 PMC7931121

[260] Donovan MG , Wren SN , Cenker M , Selmin OI , Romagnolo DF . Dietary fat and obesity as modulators of breast cancer risk: focus on DNA methylation. Br J Pharmacol. 2020;177(6):1331‐1350. 31691272 10.1111/bph.14891 PMC7056465

[261] Kanaley JA , Colberg SR , Corcoran MH , et al. Exercise/physical activity in individuals with type 2 diabetes: a consensus statement from the American College of Sports Medicine. Med Sci Sports Exerc. 2022;54(2):353‐368. 35029593 10.1249/MSS.0000000000002800 PMC8802999

[262] Tucker WJ , Fegers‐Wustrow I , Halle M , Haykowsky MJ , Chung EH , Kovacic JC . Exercise for primary and secondary prevention of cardiovascular disease: JACC Focus Seminar 1/4. J Am Coll Cardiol. 2022;80(11):1091‐1106. 36075680 10.1016/j.jacc.2022.07.004

[263] Duggan C , Tapsoba JD , Scheel J , Wang CY , McTiernan A . Weight loss reduces circulating micro‐RNA related to obesity and breast cancer in postmenopausal women. Epigenetics. 2022;17(13):2082‐2095. 35938852 10.1080/15592294.2022.2107841 PMC9665139

[264] Mastrototaro L , Roden M . The effects of extracellular vesicles and their cargo on metabolism and its adaptation to physical exercise in insulin resistance and type 2 diabetes. Proteomics. 2023:e2300078. 37525338 10.1002/pmic.202300078

[265] Trettel CDS , Pelozin BRA , Barros MP , et al. Irisin: an anti‐inflammatory exerkine in aging and redox‐mediated comorbidities. Front Endocrinol (Lausanne). 2023;14:1106529. 36843614 10.3389/fendo.2023.1106529 PMC9951776

[266] Aas V , Øvstebø R , Brusletto BS , et al. Distinct microRNA and protein profiles of extracellular vesicles secreted from myotubes from morbidly obese donors with type 2 diabetes in response to electrical pulse stimulation. Front Physiol. 2023;14:1143966. 37064893 10.3389/fphys.2023.1143966 PMC10098097

[267] Dogan S , Cicekdal MB , Ozorhan U , et al. Roles of adiponectin and leptin signaling‐related microRNAs in the preventive effects of calorie restriction in mammary tumor development. Appl Physiol Nutr Metab. 2021;46(8):866‐876. 33493087 10.1139/apnm-2020-1000

[268] Bultman SJ . A reversible epigenetic link between obesity and cancer risk. Trends Endocrinol Metab. 2018;29(8):529‐531. 29884327 10.1016/j.tem.2018.05.004

[269] Li R , Grimm SA , Mav D , et al. Transcriptome and DNA methylome analysis in a mouse model of diet‐induced obesity predicts increased risk of colorectal cancer. Cell Rep. 2018;22(3):624‐637. 29346762 10.1016/j.celrep.2017.12.071 PMC5793878

[270] Talukdar FR , Marcillo DIE , Laskar RS , et al. Bariatric surgery‐induced weight loss and associated genome‐wide DNA‐methylation alterations in obese individuals. Clin Epigenet. 2022;14(1):176. 10.1186/s13148-022-01401-9 PMC9759858 36528638

[271] Zhang LH , Wang J , Tan BH , Yin YB , Kang YM . The association of lncRNA and mRNA changes in adipose tissue with improved insulin resistance in type 2 obese diabetes mellitus rats after Roux‐en‐Y gastric bypass. Dis Markers. 2022;2022:8902916. 35899178 10.1155/2022/8902916 PMC9313968

[272] Aminian A , Wilson R , Al‐Kurd A , et al. Association of bariatric surgery with cancer risk and mortality in adults with obesity. JAMA. 2022;327(24):2423‐2433. 35657620 10.1001/jama.2022.9009 PMC9166218

[273] Breininger SP , Sabater L , Malcomson FC , Afshar S , Mann J , Mathers JC . Obesity and Roux‐en‐Y gastric bypass drive changes in miR‐31 and miR‐215 expression in the human rectal mucosa. Int J Obes (Lond). 2021;46(2):333‐341. 10.1038/s41366-021-01005-y PMC8794786 34716428

[274] Blum A , Yehuda H , Geron N , Meerson A . Elevated levels of miR‐122 in serum may contribute to improved endothelial function and lower oncologic risk following bariatric surgery. Isr Med Assoc J. 2017;19(10):620‐624. 29103239

[275] Fang Z , Fan M , Yuan D , et al. Downregulation of hepatic lncRNA Gm19619 improves gluconeogenesis and lipogenesis following vertical sleeve gastrectomy in mice. Commun Biol. 2023;6(1):105. 36707678 10.1038/s42003-023-04483-0 PMC9883214

[276] Wei W , Wang X , Wei Y , et al. lncRNA TUG1 protects intestinal epithelial cells from damage induced by high glucose and high fat via AMPK/SIRT1. Mol Med Rep. 2022;25(4):139. 10.3892/mmr.2022.12655 35211764

[277] Hosseini H , Teimouri M , Shabani M , et al. Resveratrol alleviates non‐alcoholic fatty liver disease through epigenetic modification of the Nrf2 signaling pathway. Int J Biochem Cell Biol. 2020;119:105667. 31838177 10.1016/j.biocel.2019.105667

[278] Liu DB , Wong CC , Fu L , et al. Squalene epoxidase drives NAFLD‐induced hepatocellular carcinoma and is a pharmaceutical target. Sci Transl Med. 2018;10(437):eaap9840. 10.1126/scitranslmed.aap9840 29669855

[279] Yan F , Shen N , Pang JX , et al. Fatty acid‐binding protein FABP4 mechanistically links obesity with aggressive AML by enhancing aberrant DNA methylation in AML cells. Leukemia. 2017;31(6):1434‐1442. 27885273 10.1038/leu.2016.349 PMC5457366

[280] Andrade FO , Nguyen NM , Warri A , Hilakivi‐Clarke L . Reversal of increased mammary tumorigenesis by valproic acid and hydralazine in offspring of dams fed high fat diet during pregnancy. Sci Rep. 2019;9(1):20271. 31889127 10.1038/s41598-019-56854-5 PMC6937280

[281] Suárez R , Chapela SP , Álvarez‐Córdova L , et al. Epigenetics in obesity and diabetes mellitus: new insights. Nutrients. 2023;15(4):811. 10.3390/nu15040811 PMC9963127 36839169

[282] Ringel AE , Drijvers JM , Baker GJ , et al. Obesity shapes metabolism in the tumor microenvironment to suppress anti‐tumor immunity. Cell. 2020;183(7):1848‐1866. e26. 33301708 10.1016/j.cell.2020.11.009 PMC8064125

[283] Sanchez‐Pino MD , Gilmore LA , Ochoa AC , Brown JC . Obesity‐associated myeloid immunosuppressive cells, key players in cancer risk and response to immunotherapy. Obesity (Silver Spring). 2021;29(6):944‐953. 33616242 10.1002/oby.23108 PMC8154641

[284] Xiong Z , Li X , Yang L , et al. Integrative analysis of gene expression and DNA methylation depicting the impact of obesity on breast cancer. Front Cell Dev Biol. 2022;10:818082. 35350384 10.3389/fcell.2022.818082 PMC8957964

[285] Woodall MJ , Neumann S , Campbell K , Pattison ST , Young SL . The effects of obesity on anti‐cancer immunity and cancer immunotherapy. Cancers (Basel). 2020;12(5):1230. 10.3390/cancers12051230 PMC7281442 32422865

[286] Sharma BR , Kanneganti TD . NLRP3 inflammasome in cancer and metabolic diseases. Nat Immunol. 2021;22(5):550‐559. 33707781 10.1038/s41590-021-00886-5 PMC8132572

[287] Zhang L , Li H , Zang Y , Wang F . NLRP3 inflammasome inactivation driven by miR‑223‑3p reduces tumor growth and increases anticancer immunity in breast cancer. Mol Med Rep. 2019;19(3):2180‐2188. 30747211 10.3892/mmr.2019.9889 PMC6390045

[288] Chakravarti R , Lenka SK , Gautam A , et al. A review on CRISPR‐mediated epigenome editing: a future directive for therapeutic management of cancer. Curr Drug Targets. 2022;23(8):836‐853. 35078394 10.2174/1389450123666220117105531

[289] Goell JH , Hilton IB . CRISPR/Cas‐based epigenome editing: advances, applications, and clinical utility. Trends Biotechnol. 2021;39(7):678‐691. 33972106 10.1016/j.tibtech.2020.10.012

[290] Feinberg AP , Levchenko A . Epigenetics as a mediator of plasticity in cancer. Science. 2023;379(6632):eaaw3835. 36758093 10.1126/science.aaw3835 PMC10249049

[291] Abdulhaq H , Rossetti JM . The role of azacitidine in the treatment of myelodysplastic syndromes. Expert Opin Investig Drugs. 2007;16(12):1967‐1975. 10.1517/13543784.16.12.1967 18042004

[292] Atallah E , Kantarjian H , Garcia‐Manero G . The role of decitabine in the treatment of myelodysplastic syndromes. Expert Opin Pharmacother. 2007;8(1):65‐73. 17163808 10.1517/14656566.8.1.65

[293] Hoy SM . Tazemetostat: first approval. Drugs. 2020;80(5):513‐521. 32166598 10.1007/s40265-020-01288-x

[294] Keam SJ . Valemetostat tosilate: first approval. Drugs. 2022;82(16):1621‐1627. 36380144 10.1007/s40265-022-01800-5 PMC9705507

[295] Siegel D , Hussein M , Belani C , et al. Vorinostat in solid and hematologic malignancies. J Hematol Oncol. 2009;2:31. 19635146 10.1186/1756-8722-2-31 PMC2731787

[296] Sawas A , Radeski D , O'Connor OA . Belinostat in patients with refractory or relapsed peripheral T‐cell lymphoma: a perspective review. Ther Adv Hematol. 2015;6(4):202‐208. 26288714 10.1177/2040620715592567 PMC4530372

[297] Smolewski P , Robak T . The discovery and development of romidepsin for the treatment of T‐cell lymphoma. Expert Opin Drug Discov. 2017;12(8):859‐873. 28641053 10.1080/17460441.2017.1341487

[298] Shi Y , Jia B , Xu W , et al. Chidamide in relapsed or refractory peripheral T cell lymphoma: a multicenter real‐world study in China. J Hematol Oncol. 2017;10(1):69. 28298231 10.1186/s13045-017-0439-6 PMC5351273

[299] Laubach JP , Moreau P , San‐Miguel JF , Richardson PG . Panobinostat for the treatment of multiple myeloma. Clin Cancer Res. 2015;21(21):4767‐4773. 26362997 10.1158/1078-0432.CCR-15-0530

[300] Donker ML , Ossenkoppele GJ . Evaluating ivosidenib for the treatment of acute myeloid leukemia. Expert Opin Pharmacother. 2020;21(18):2205‐2213. 32808831 10.1080/14656566.2020.1806822

[301] Stein EM , DiNardo CD , Pollyea DA , et al. Enasidenib in mutant IDH2 relapsed or refractory acute myeloid leukemia. Blood. 2017;130(6):722‐731. 28588020 10.1182/blood-2017-04-779405 PMC5572791

